# Use of ambulatory pathways in emergency general surgery: a systematic review

**DOI:** 10.1136/bmjopen-2025-099203

**Published:** 2025-09-28

**Authors:** Benjamin Fox, Mary Walters, Samir Pathak, Adam Peckham-Cooper, Natalie S Blencowe

**Affiliations:** 1 Leeds Teaching Hospitals NHS Trust, Leeds, UK; 2 NHS Greater Glasgow and Clyde, Glasgow, UK; 3 Mid Yorkshire Hospitals NHS Trust, Wakefield, UK; 4 University of Leeds, Leeds, UK; 5 Centre for Surgical Research, Bristol Medical School, Bristol, UK

**Keywords:** SURGERY, Safety, Delivery of Health Care, Integrated, Systematic Review

## Abstract

**Abstract:**

**Objectives:**

Ambulatory care is defined as the provision of medical treatment by healthcare professionals outside an inpatient hospital setting. While well-established in acute medicine, uptake of ambulatory pathways in emergency general surgery (EGS) is variable and optimal design and delivery is unclear in this context. This systematic review sought to (1) appraise current EGS ambulatory pathway literature and (2) ascertain the constituent components across the identified pathways, guiding the development of comprehensive templates for future EGS ambulatory pathways.

**Design:**

Systematic review.

**Data sources:**

PubMed, Embase, Medline and Cochrane Library, from 5 December 2018 to 5 December 2023 inclusive.

**Eligibility criteria:**

All primary observational studies (ie, case–control, cohort studies and randomised controlled trials (RCTs)) were included. Case series and conference abstracts were excluded due to the high likelihood of incomplete data. Studies reporting paediatric or non-surgical populations, or ambulatory surgical care within a primary care setting, were also excluded.

**Data extraction and synthesis:**

General study characteristics (year and journal of publication, country of origin, study design, disease area, number of patients receiving ambulatory management and use of control groups) were recorded. To identify the constituent components of EGS ambulatory pathways, an initial subset of five papers was reviewed, from which four categories were identified (decision-making processes, scoring/classification systems, investigations and care escalation and discharge criteria). An additional fifth component (‘follow-up’) was identified during data extraction. Reporting of the constituent components of ambulatory pathways was also extracted, as well as outcomes including readmission, complications and mortality.

**Results:**

Of 43 included studies, there were 8 RCTs, 31 cohort studies and 4 studies using other methods. Reporting of all aspects of EGS ambulatory pathways was heterogeneous. 24 (56%) papers reported the specialty and grade of clinician acting as senior decision-maker. 17 different scoring/classification systems were used. 32 (74%) papers described using investigations to select ambulatory patients, including blood tests (n=12) and imaging (n=16). Eight studies (19%) specified both care escalation and discharge criteria. Information about follow-up was described in 29 papers, with location (n=29), time points (n=26), personnel (n=16) and the form of the follow-up (n=23) all reported variably. Readmission rates were recorded in 34 studies and ranged from 0% to 13%. Most studies (n=32) reported 30-day readmission, although 48 hours (n=1) and 90 days (n=1) were also used. Mortality was recorded in 24 papers, with 21 reporting a mortality rate of 0 and the remaining 3 reporting rates of <0.5%.

**Conclusions:**

Key components of published EGS ambulatory pathways include decision-making processes, scoring/classification systems, investigations, care escalation and discharge criteria, and follow-up. However, this information is currently inconsistently reported. Future work to identify and agree on guidelines for the ‘core’ components of ambulatory EGS pathways is needed, to facilitate cross-study comparisons, and crucially, provide a ‘gold-standard’ framework for developing future ambulatory pathways.

**STRENGTHS AND LIMITATIONS OF THIS STUDY**

- 43 studies were included in the systematic review, encompassing more than 2.6 million patients.
- Each study was assessed in granular detail to compare constituent components of each ambulatory pathway.
- Methods were robust and included clearly stated inclusion and exclusion criteria.
- Limitations of the study included the need to include level III evidence and below, and the heterogeneity of conditions investigated across included studies.

## Introduction

 Ambulatory care can be defined as the provision of medical care by healthcare professionals outside an inpatient hospital setting.^1^ Initially implemented within emergency departments (EDs) and general medicine, there is a desire to emulate this success in the emergency general surgical (EGS) setting.^2^ The Association of Surgeons of Great Britain and Ireland’s Emergency General Surgery commissioning guide (2014) recommends the use of ambulatory pathways for patients presenting with biliary colic, cholecystitis, diverticulitis and non-specific abdominal pain.^3^ However, detailed pathways were not provided, and uptake has been slow, with variable engagement from surgical units across the UK.

The primary focus of an ambulatory pathway is to provide equivocal standards of care for patients outwith the hospital setting.^4^ Key criteria, such as clear discharge and readmission parameters, may help to minimise unplanned rehospitalisation of patients on the ambulatory pathway; for example, enabling identification of ‘high-risk’ patients in whom ambulatory care may not be appropriate. Commonly, a prompt initial clinical review is undertaken to enable early decisions to be made regarding ambulation. After determining suitability for ambulation, patients are discharged with safety netting advice and medication if required, with a clear follow-up plan. Recent guidance from Getting it Right First Time (GIRFT) has reinforced the need for early input from ‘senior decision-makers’ (SDMs) and for timely reporting of imaging studies, to facilitate early discharge where possible.^5^ Studies have shown this ‘front door’ input can reduce emergency admissions by 20%–30%,^6 7^ facilitate more effective prioritisation of clinically unwell patients, while conferring financial efficiencies.^8 9^

When developing EGS ambulatory pathways, both patient and institutional factors require consideration and it is essential that a robust, systematic assessment of the pathway should be undertaken prior to wide implementation, with a particular focus on feasibility and safety. Poorly delivered and designed ambulatory pathways may compromise patient outcomes and demand high levels of staffing, potentially relocating medical staff away from other emergency care facilities.^3 10^ Currently, however, the optimal design, delivery and evaluation of ambulatory pathways and services remains unclear. In addition, there is a distinct lack of literature on the definition of an ambulatory pathway, as well as its key components. The aims of this study are, therefore, to (1) systematically summarise and appraise current literature regarding ambulatory pathways for general surgical pathologies/conditions and (2) ascertain the constituent components across the identified pathways, to guide the development of comprehensive templates enabling future pathway design.

## Methods

This review was performed in accordance with the Preferred Reporting Items for Systematic Reviews and Meta-Analyses guidelines.^11^

### Search strategy and study identification

Systematic searches—including terms for ambulatory care, general surgery and specific surgical conditions—were undertaken in PubMed, Embase, Medline and Cochrane Library, to include studies dating from 5 December 2018 to 5 December 2023 inclusive (online supplemental appendix 1 and 2).

### Study eligibility

Primary observational studies (ie, case–control, cohort studies) and randomised controlled trials (RCTs) providing data about EGS ambulatory pathways were included. Case series and conference abstracts were excluded due to the high likelihood of incomplete data. Studies reporting paediatric or non-surgical populations, or ambulatory surgical care within a community care setting (such as by general practitioners/family doctors) were also excluded. Non-English language studies were excluded.

### Study selection

Following removal of duplicates, titles and abstracts were screened by two independent reviewers (BF and MW). Subsequently, full texts were retrieved and screened for eligibility in the same manner using a software programme, Rayyan.^12^ Conflicts at both stages were resolved by discussion involving a third independent reviewer (AP-C/SP/NSB) to provide a final list of included papers.

### Data extraction

Data extraction was performed independently by two reviewers (BF and MW), within the following categories.

#### Characteristics of included studies

The year and journal of publication, country of origin, study design (including temporality, ie, whether prospective or retrospective), and use of control groups (if applicable) were recorded. The total number of patients in each study, numbers entering ambulatory pathways, the number of participating centres, and the disease process(es) or treatments (whether surgical or non-surgical) of interest were also collected.

#### Ambulatory pathway components

Reporting of the constituent components of ambulatory pathways was assessed. Initially, a subset of five papers was reviewed from which four categories were identified: (1) decision-making processes (eg, seniority of decision-maker(s) and the time point of decisions), (2) scoring/classification systems, (3) investigations and (4) discharge and readmission criteria. This was an iterative process, and where subsequent papers reported further information, additional categories were created.

#### Outcomes

The number/proportion of patients successfully completing an ambulatory pathway was recorded. Any reasons provided for non-completion were extracted and summarised. Outcomes including complications, care escalation to hospital and mortality were collected. Care escalation was defined as any inpatient stay after discharge onto an ambulatory pathway. Dependent on the pathway, this may have occurred preoperatively or postoperatively or may not have been associated with an operative procedure.

#### Comparison of pathways

Where more than five included studies described ambulatory pathways for the same surgical condition, reporting of the components of these pathways was compared, to highlight consistencies and discrepancies.

### Data synthesis and analysis

Data were summarised using a narrative synthesis and where relevant, descriptive statistics.

### Risk of bias assessment

For included RCTs, Risk of Bias assessments were performed by two reviewers (BF and MW) using the Cochrane Risk of Bias 2 tool.^13^ Discrepancies were discussed and resolved by a third reviewer (NSB).

### Patient and public involvement

Patients and the public were not involved in the design, conduct, reporting or dissemination plans of this secondary research study.

## Results

**Figure 1:**
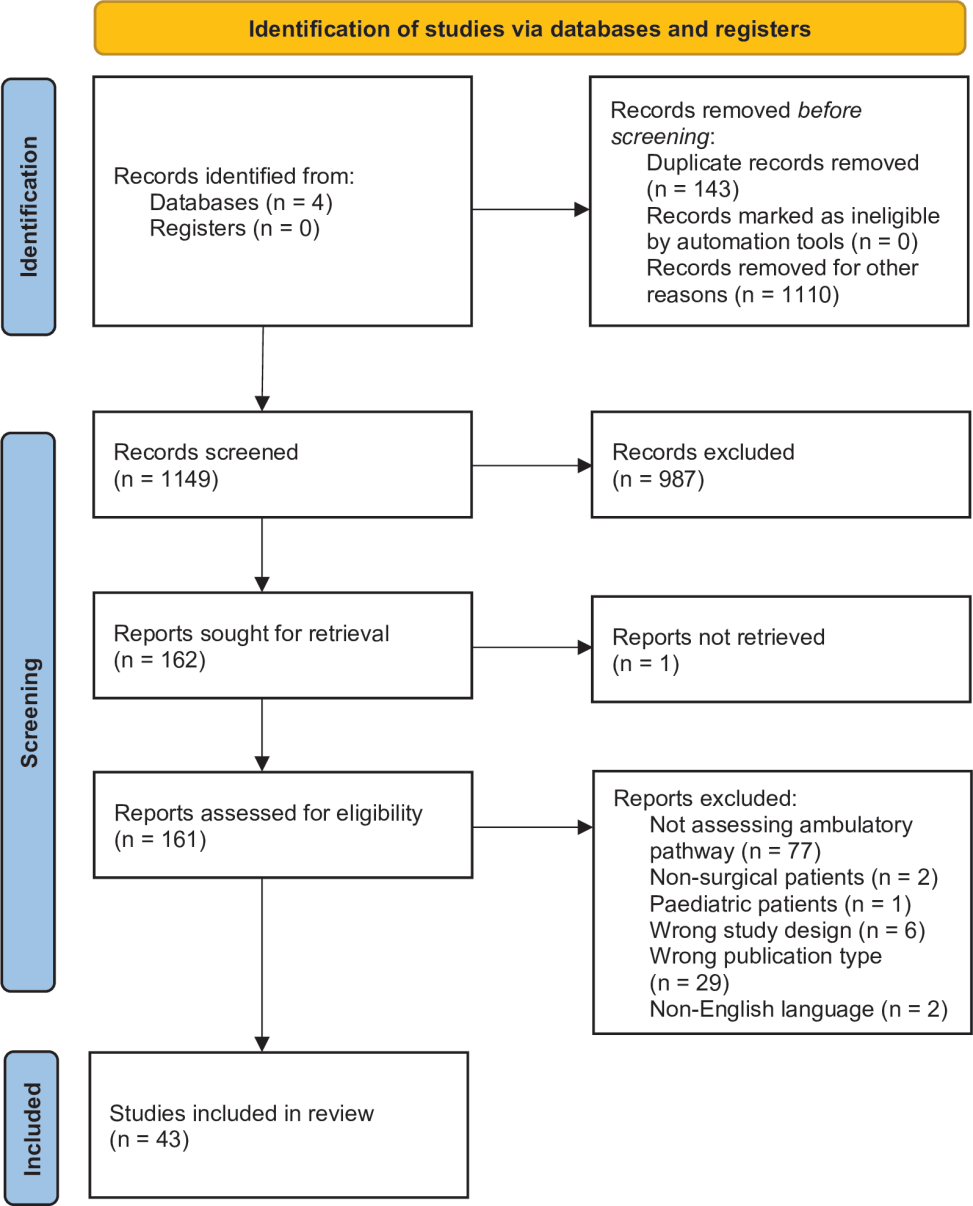
PRISMA flow chart displaying the decision-making process that resulted in final study selection. PRISMA, Preferred Reporting Items for Systematic Reviews and Meta-Analyses.

Searches identified 1149 titles/abstracts, of which 987 were excluded, leaving 162 articles for full text screening. 118 were excluded and 1 paper could not be found, leaving a total of 43 articles for analysis (figure 1).

### Characteristics of included studies

Of the 43 included studies, there were 8 RCTs, 31 cohort studies (25 retrospective, 6 prospective) and 4 studies using other methods (2 mixed methods, 1 retrospective cross-sectional, 1 retrospective case–control) (table 1). A total of 2 635 243 patients were included across the studies, of which 1 381 359 (52%) were on ambulatory pathways (the remainder did not meet the criteria for ambulatory management). Most studies were single-centre (n=29) and conducted in Europe (n=25) (online supplemental appendix 3). The most studied conditions were herniae (n=10); biliary disease (including biliary colic, common bile duct stones and acute cholecystitis (n=8)), appendicitis (n=7) and diverticulitis (n=6). Six of the studies included multiple surgical conditions. Three RCTs compared ambulatory pathways with standard inpatient care, while the other five compared various aspects of ambulatory pathways including different surgical methods (n=2), antibiotic regimes (n=2) and the use of preoperative education (n=1).

**Table 1:** General study characteristics

	Number of studies n=43 (%)
Year of publication	
2018–2020	17 (40)
2021–2023	26 (60)
Study design	
RCT	8 (19)
Cohort study	31 (72)
Other	4 (2)
Temporality of data collection	
Retrospective	28 (65)
Prospective	7 (16)
Not reported	8 (19)
Number of centres	
Single	29 (67)
Multiple	14 (33)
Study location	
Africa	2 (5)
Asia	2 (5)
Australia	4 (9)
Europe	26 (60)
North America	9 (21)
Disease process	
Abscess	2 (5)
Appendicitis	7 (16)
Biliary	8 (19)
Diverticular disease	6 (14)
Herniae	10 (23)
Pancreatitis	1 (2)
Multiple disease	6 (14)
Other	3 (7)
Study population	
0–99	8 (19)
100–199	8 (19)
200–299	5 (12)
300–399	2 (4)
400–499	2 (4)
500–999	5 (12)
>1000	13 (30)
Number of patients on ambulatory pathway	
0–99	16 (37)
100–199	6 (14)
200–299	5 (12)
300–399	5 (12)
400–499	2 (5)
500–999	1 (2)
>1000	8 (19)

RCT, randomised controlled trial.

### Ambulatory pathway components

An additional component ‘follow-up’ was identified during data extraction, bringing the total number of ambulatory components to five. While ‘imaging’ was also identified, this was incorporated into the pre-existing category ‘investigations’. Reporting of ambulatory pathway components is summarised below and within online supplemental appendix 4.

#### Decision-making processes

In 11 papers, review by a SDM was described as an integral part of the ambulatory pathway (ie, ensuring appropriate enrolment of patients and use of the pathway itself), whereas this was unclear in the remaining studies and none of the included papers provided a definition of the role of the SDM. 24 (56%) reported the specialty and grade of clinician acting as SDM. The SDM was most commonly a consultant/attending (EGS/general surgery=6, ED=3, anaesthetics=2), while in three studies the SDMs were EGS/General Surgery residents (n=3).

Six of the 43 studies commented on the specific timing of ambulation. In two, ambulation occurred postoperatively (within 6–8 hours), one within 8 hours of treatment in the ED, one following an ultrasound scan within 24 hours, one within 72 hours following joint consultation between ED and general surgery, and one following a scheduled appointment with the EGS team (the timing of which was not specified).

#### Scoring/classification systems

26 of the papers described using at least one scoring and/or classification system. Across these, 17 different systems were used, for a variety of purposes: disease severity/diagnostic workup (n=6), comorbidities (n=3), patient demographics (n=1), suitability for ambulation/discharge (n=3), postoperative outcomes (n=3) and symptom assessment (n=1). Six of the scoring/classification systems were disease-specific (modified Hinchey classification, modified Neff classification, Appendicitis Inflammatory Response (AIR) score, Alvarado score, St Antoine’s score, Tokyo Guidelines 2013/2018) and the rest were generic (table 2).

**Table 2:** Scoring and classification systems used in studies

Scoring/classification system		Studies using system (n)	Description of use
Generic	Clavien Dindo classification	9	Assessment of patient outcomes
	Visual Analogue Scale	8	Assessment of symptoms
	Aldrete Score	6	Discharge criteria
	Charlson Comorbidity Index	3	Assessment of patient comorbidities
	Comprehensive Complication Index	2	Assessment of patient outcomes
	Elixhauser Comorbidity Score	1	Assessment of patient comorbidities
	EuroQol 5 Dimension Score	1	Assessment of patient outcomes
	French High Health Authority score (G)	1	Assessment of disease severity
	Hospital Frailty Risk Score (G)	1	Assessment of patient comorbidities
	Index of Multiple Deprivation score (G)	1	Assessment of patient demographics
	PADSS (G)	1	Discharge criteria
Disease specific	Modified Hinchey Classification	4	Assessment of disease severity
	Tokyo Guidelines 2013 and 2018 (G)	1	Assessment of disease severity
	St Antoine’s Score	3	Assessment of patient suitability for ambulation
	Alvarado Score	2	Diagnostic workup
	Appendicitis Inflammatory Response score	1	Diagnostic workup
	Modified Neff classification (DS)	1	Assessment of disease severity

PADSS, Post Anaesthetic Discharge Scoring System.

#### Investigations

Of the 41 papers, 32 (74%) described the use of investigations to select patients for ambulation, including blood tests (n=12) and imaging (n=16). The most common blood tests used were full blood count (n=12), CRP (n=7), and urea and electrolytes (n=6). The most common forms of imaging were CT (n=15) and ultrasound (n=11).

#### Care escalation/discharge criteria

Eight studies (19%) specified both care escalation and discharge criteria, with an additional 11 papers specifying only care escalation (n=1) or discharge (n=10) criteria. The most common criteria for discharge were pain control (n=10) and adequate oral intake (n=9), while the most common care escalation criteria were fever (n=5) and pain (n=4). A total of 19 different discharge and 17 care escalation criteria were identified across the studies. No consistent criteria for either care escalation or discharge were used across all studies (table 3).

**Table 3:** Reporting of care escalation and discharge criteria

		Number of papers n=43 (%)
Discharge criteria	Adequate pain control	10 (23)
	Adequate oral intake	9 (21)
	Stable vital signs/haemodynamics	6 (14)
	Absence of nausea and vomiting	4 (9)
	Uncomplicated disease	4 (9)
	Aldrete criteria	4 (9)
	Micturition	3 (7)
	Patient agreement	3 (7)
	Absence of fever	3 (7)
	Ability to ambulate	3 (7)
	Good social support	2 (5)
	Follow-up organised	2 (5)
	Adequate consciousness level	2 (5)
	Absence of bleeding	2 (5)
	Acceptable inflammatory markers	1 (2)
	Absence of rebound tenderness	1 (2)
	Lack of significant comorbidities	1 (2)
	Lack of complications	1 (2)
	PADSS	1 (2)
	Not reported	25 (58)
Readmission criteria	Fever	5 (12)
	Pain	4 (9)
	Nausea and vomiting	3 (7)
	Clinical worsening	4 (9)
	Abdominal distension	2 (5)
	Wound dehiscence	1 (2)
	Bleeding	1 (2)
	Surgical site swelling	1 (2)
	Jaundice	1 (2)
	Poor symptomatic control	1 (2)
	Rising inflammatory markers	1 (2)
	Abnormal observations	1 (2)
	Unable to maintain self-care	1 (2)
	Unable to maintain oral intake	1 (2)
	Generally unwell	1 (2)
	Concerning CT findings	1 (2)
	Not reported	34 (79)

PADSS, Post Anaesthetic Discharge Scoring System.

#### Follow-up

Follow-up was described in 29 papers, with location (n=29), time points (n=26), personnel (n=16) and the form that the follow-up took (n=23) reported variably. Most commonly, follow-up took place in an ambulatory clinic (n=21) or remotely using telehealth (n=9) and was clinician-led (n=14) using history and examination (n=23). Six studies also described ‘ongoing management’, which included continued antibiotic prescriptions and surgical planning. Follow-up time points were variable, with some studies reviewing patient’s multiple times (table 4).

**Table 4:** Reporting of follow-up of patients following ambulatory pathway

		Number of studies n=43 (%)
Follow-up location	Ambulatory clinic	21 (49)
	Telephone	9 (21)
	Patient’s home	3 (7)
	Emergency department	2 (5)
	Surgical assessment unit	1 (2)
	General practitioner	1 (2)
	Not reported	15 (35)
Personnel undertaking follow-up appointment	Nurse	6 (14)
	Doctor	14 (33)
	Other	2 (5)
	Not reported	27 (63)
Format of follow-up appointment	History and examination	23 (53)
	Bloods	6 (14)
	Imaging	6 (14)
	Ongoing management	6 (14)
	Not reported	18 (42)
Time point of follow-up	Day 1	10 (23)
	Day 2	7 (16)
	Day 3	5 (12)
	Day 4	1 (2)
	Day 7	7 (16)
	2 weeks	1 (2)
	1 month	12 (28)
	4–8 weeks	3 (7)
	3 months	2 (5)
	1 year	1 (2)
	Not reported	20 (47)
Number of follow-up encounters	1	10 (23)
	2	5 (12)
	3	3 (7)
	4	5 (12)
	Not reported	20 (47)

### Postoperative outcomes

Readmission rates were recorded in 34 studies and ranged from 0% to 13%. Most studies (n=32) reported 30-day readmission, although 48 hours (n=1) and 90 days (n=1) were also used. Mortality was recorded in 24 papers, with 21 reporting a mortality rate of 0 and the remaining 3 reporting rates of <0.5%. None of the three papers that reported a mortality rate greater than 0 described the cause of the mortality.

Complication rates were recorded in 34 studies and ranged from 0% to 48.5%. Eight of these studies reported ‘complications’ but did not provide further details. In the remainder, reported complications varied between studies and included ongoing symptoms such as postoperative pain (n=10) and uncontrolled nausea and vomiting (n=6), as well as surgical site infections (n=9), urinary retention (n=7), abscess (n=5), haemorrhage (n=5) and intraoperative complications (n=5).

### Comparison of pathways

Similarities and differences in reporting of the key ambulatory pathway components across the most studied conditions are provided in online supplemental appendix 5. Reporting of ambulatory components was variable within studies reporting on the same condition. For example, across the seven studies reporting on appendicitis, both Alvarado (n=2) and AIR (n=1) were used for ‘scoring’ purposes (with the remainder not using a scoring system) and three studies used the St Antoine’s score to determine suitability for ambulation (with the remainder not assessing suitability for ambulation). In the six studies reporting on ambulation in diverticulitis, modified Hinchey (n=4) and Neff (n=1) scores were used to classify severity (with the remainder not categorising severity). No studies describing the ambulatory management of hernias reported symptom severity and in the eight studies of biliary disease, just one recorded severity using the Tokyo Guidelines.

### Risk of bias assessment

Of the eight RCTs, five were considered at ‘high risk’ of bias (the most common areas of concern were missing outcome data and outcome measurement) with 1 having ‘some concerns’ and 2 being ‘low risk’ (online supplemental appendix 6).

## Discussion

This review summarised current evidence regarding the ambulatory management of EGS conditions and identified five key pathway components: decision-making processes, classification systems, investigations, care escalation/discharge criteria and follow-up. Among the 43 included papers, reporting of these components was variable and there was little agreement on which outcomes should be measured (and how). Key components of ambulatory pathways—for example, six studies commented on the timing of the decision to ambulate a patient—were not considered, leaving ambiguity as to the optimal time for patients to enter an ambulatory pathway. Reporting of complications (n=34) and mortality (n=24), either preoperatively or postoperatively, and when these occurred within each specific ambulatory pathway, was also not adequately described. In addition, only eight studies reported adequate care escalation and discharge criteria, leaving significant room for error when making decisions regarding a patient’s course within a specific ambulatory pathway. There is, therefore, an urgent need for a clear, structured approach to detailing the essential components of a pan-pathology surgical ambulatory pathway.

Ambulation in the management of acute medical conditions is better established than in surgery. While this was initially sporadic and focused on specific conditions (eg, influenza, pneumonia and cellulitis),^14^ it has since expanded rapidly, resulting in the publication of national guidance (the SAMEDAY strategy) in 2024 to develop and standardise ambulatory care pathways.^15^ Guidance includes the need for a senior clinical decision-maker, clear inclusion and exclusion criteria, and suggested metrics for service evaluation. This is supported by recent GIRFT guidance, which defines the role of a SDM as ‘those who can collate relevant patient information, make a diagnosis and determine a management plan’.^5^ Based on this progress, similar steps are required to promote greater consistency and transparency in the development and implementation of surgical ambulatory pathways.

Previous reviews have attempted to evaluate the commonly reported quality indicators in ambulatory surgery.^16^ These indicators have often been divided into ‘preoperative’ (which include same-day cancellation and cancellation of procedures after patient arrival), ‘perioperative’ (including clinical information provided) and ‘postoperative’ (which include 24 hours postoperative clinical state, recovery quality (degree of pain and fatigue) and patient satisfaction).^16^ However, these quality indicators are not applicable to all ambulatory pathways, given that many EGS conditions (eg, diverticulitis, pancreatitis) do not require surgery. This study has promoted the development of more universally recognised quality indicators: decision-making processes, classification systems, investigations, care escalation/discharge criteria and follow-up. We propose building on these elements into a blueprint for the ‘gold-standard’ development of an ambulatory pathway, to include an overarching ‘core set’ of ambulatory pathway components, that should always be included and reported. Facilitating an agreed structure would help to promote safety and improve the quality and consistency of future pathways, facilitating cross-study comparisons between centres and pathways. This ‘core set’ of components could include agreed definitions on the role, level and purpose of a SDM, universal discharge criteria, and measures of success and failure (table 5). Application of pre-existing generic classifications (both of severity and suitability for ambulation) could also improve consistency in reporting. National recommendations to embed the Emergency Care Data Standard—a national project designed by the Royal College of Emergency Medicine to promote comprehensive patient data collection on presentation, diagnosis, discharge and follow-up—into the designing of ambulatory pathways^17^ may go some way to helping this reporting. This will help standardise the data collected on admission to the ED, which is the most likely first clinical interaction ambulatory pathway patients will experience.

**Table 5:** Summary of suggested ‘Core Set’ of descriptors for construction of ambulatory pathway

SDM	Scoring and classification systems	Investigations	Care escalation/discharge criteria	Follow-up
Agreed role of SDM within ambulatory pathway Agreed level of experience sufficient to undertake role of SDM Clear aims of SDM with regard to ambulation. This could include whether the SDM is also the final decision-maker in the ambulatory process	Use of a minimum number of risk severity scoring systems to complement decisions regarding ambulation. For example, use of HAPS score in acute pancreatitis Clear cut-offs for when ambulation of a patient is considered too high-risk Built-in use of ‘clinical judgement’ to complement severity scoring, to ensure we are not just ‘treating numbers’	Appropriate and timely use of imaging such as Ultrasound or CT, including whether this is used to determine suitability for ambulation Follow-up investigations, including repeat blood tests, are a mandatory part of the ongoing review of patients on an ambulatory pathway A second pathway for those patient’s who’s imaging requires non-ambulatory management, to ensure the same standards of care are maintained for both ambulatory and non-ambulatory patients	Clear, defined criteria for care escalation of patients following regular clinic follow-up Consider the use of physical, biochemical and radiological criteria to ensure a full clinical picture is used to make decisions regarding ambulation Discharge criteria that risk-stratify patients to ensure that patients who are ambulated are suitable for ambulation Complications resulting in re-admission should be documented and scoring systems (such as Clavien-Dindo) should be used to risk-stratify these complications. These can form a basis for identifying areas for improvement within the pathway itself	Use of novel resources, for example, remote virtual monitoring wards to identify early those patients who are at high risk and who may require non-ambulatory care Consider early, regular follow-up of patients on ambulatory pathways, and clear pathways for surgical intervention should this be necessary (eg, use of ‘hot’ gallbladder lists for ambulated patients who require laparoscopic cholecystectomy)

HAPS, Harmless Acute Pancreatitis Score; SDM, senior decision-maker.

Although this review is novel, there are limitations. First, the authors presumed that a lack of reporting meant that the component of the pathway did not exist, which may represent an over-simplification. Second, the review intended to give an overarching view of ambulatory surgical care and, therefore, included many different surgical conditions, which may have influenced the lack of consistency in results, although ‘matched’ comparisons of papers reporting pathways for the same condition suggest this is not the case. The variety in surgical conditions included in this review meant that performing a meta-analysis would have had limited value. A third limitation is that we did not include studies about ambulatory pathways in other areas such as EDs and general medicine. This may be an important omission as their pathways are likely to be more developed, and therefore, summarising the components of such pathways is an urgent area of future research. The authors also recognise that while a Risk of Bias (ROB) assessment tool was used for the randomised control trials, quality assessment was not undertaken for other study designs. Given most of the included RCTs were at high ROB, we anticipated that the quality of the other studies would also be poor. Finally, only English language studies were included, meaning other healthcare systems may have been missed.

Ambulatory surgical care is a rapidly evolving field aligned with the evolution of EGS in the UK. There is huge potential for improvements to be made in ambulatory surgical pathways across the spectrum of surgical specialties and pathologies. This study found that currently, reporting of ambulatory general surgical pathways is inconsistent. Future work should focus on a ‘core set’ of ambulatory surgical pathway components, to facilitate comparison of pathways and pave the way for development of a standardised, safe and effective template for surgical ambulation.

## Supplementary material

10.1136/bmjopen-2025-099203 online supplemental file 1

## Data Availability

Data are available on reasonable request.
