# Correction: Use of ambulatory pathways in emergency general surgery: a systematic review

**DOI:** 10.1136/bmjopen-2025-099203corr1

**Published:** 2026-05-27

Fox B, Walters M, Pathak S *et al.* Use of ambulatory pathways in emergency general surgery: a systematic review. *BMJ Open* 2025;15:e099203. doi:10.1136/bmjopen-2025-099203

This article was previously published with an error.

The funding statement missed the MRC funder (grant number: MR/S001751/1).

