# Supplementary material for: Use of ambulatory pathways in emergency general surgery: a systematic review
Source: BMJ Open. 2025 Sep 28;15(9):e099203. doi: 10.1136/bmjopen-2025-099203 (PMC12481336; doi:10.1136/bmjopen-2025-099203)
Supplement: online supplemental file 1 [file bmjopen-15-9-s001.docx]

**A Systematic Review of the use of Ambulatory Pathways in Emergency General Surgery**

**Category**

Systematic Review

**Authors**

*Mr Benjamin J Fox^1,4^ Speciality Registrar (ST2) - Trauma and Orthopaedic Surgery

*Dr Mary Walters^1,2^ Foundation Year Doctor

Mr Samir Pathak^1,5^ Consultant Pancreatic Surgeon

Mr Adam Peckham-Cooper^1,5^ Consultant Emergency General Surgeon

Ms Natalie S Blencowe^1,3^ Consultant Emergency General Surgeon and Associate Professor of Surgery

* These authors contributed equally to the study

**Affiliations**

1. Leeds Teaching Hospitals NHS Trust, Leeds, UK
2. Mid Yorkshire Hospitals NHS Trust, Wakefield, UK
3. Centre for Surgical Research, Bristol Medical School, Bristol, UK
4. NHS Greater Glasgow and Clyde, Glasgow, UK
5. University of Leeds, Leeds, UK

**Attributing Department** Leeds Emergency General Surgery Department

St James’s University Hospital

Beckett Street

Leeds

LS9 7TF

**Corresponding Author** Mr Adam Peckham-Cooper

Leeds Emergency General Surgery Department

St James’s University Hospital

Beckett Street

Leeds

LS9 7TF

E-mail: adam.peckham-cooper@nhs.net

**Supplementary Materials - Index**

|  |  |
| --- | --- |
| *Appendix 1: Search Strategy* | *Page 8* |
| *Appendix 2: PROSPERO Registration Information*  *Appendix 3: Demographics of Included Studies*  *Appendix 4: Reporting of the Components of Ambulatory Pathways across Included Papers* | *Page 8*  *Page 11*    *Page 11* |
| *Appendix 5: Comparison of Matched Pathways*  *Appendix 6: Combined Cochrane Risk of Bias 2 Tool* | *Page 14*  *Page 15* |
|  |  |
|  |  |

**Supplementary Appendixes**

**Appendix 1: Search strategy**

**Search Strategy:**

1. Search across PubMed, Embase, Medline, Web of Science, Cochrane Central Register of Controlled Trials, Cochrane Database of Systematic Reviews, and Google Scholar
2. Search keywords: (“ambulatory care” OR “ambulatory procedure” OR “outpatient” OR “day care” OR “day case” OR “day stay” OR “day surgery” OR “hot clinic” OR “office-based”) AND (“emergency general surgery” OR “EGS” OR “emergency surgery” OR “appendicitis” OR “appendectomy” OR “cholecystitis” OR “cholecystectomy” OR “diverticulitis” OR “diverticular disease” OR “abscess” OR “hernia” OR “pancreatitis”) AND (“randomised control study” OR “RCT” OR “randomised” OR “cohort study” OR “case-control study” OR “case series”)
3. Search range: from 5^th^ December 2018 to 5^th^ December 2023 inclusive
4. Identify additional articles using references of relevant studies and ‘Related articles’ link in PubMed

**Identifying/selecting articles:**

1. Independent screening of titles/abstracts by 2 authors
2. Retrieval of full text of identified relevant articles
3. Assessment of full texts for eligibility based on inclusion/exclusion criteria
4. Any disagreements – discuss between authors, if ongoing disagreement discuss with senior authors

Search 1: Search terms 1 to be combined with OR (no search restrictions)

Search 2: Search terms 2 to be combined with OR (no search restrictions)

Search 3: (Search 1) AND (Search 2) – restricted to English language, last 5 years, full-text articles only

**Search 1:**

| **MeSH terms** | **MeSH subheadings to include** | **MeSH subheadings not included** | **Free text search terms and synonyms** |
| --- | --- | --- | --- |
| Ambulatory Care |  | Peritoneal dialysis, continuous ambulatory |  |
| Ambulatory Surgical Procedure |  |  |  |
| Outpatients |  |  | Outpatient |
|  |  |  | Out-patient |
|  |  |  | Out-patients |
| Day care, medical |  |  | Day care |
| Day surgery |  |  |  |
|  |  |  | Day case |
|  |  |  | Hot clinic |
|  |  |  | Office based |
|  |  |  | Office-based |

**Search 2:**

| **MeSH terms** | **MeSH subheadings to include** | **MeSH subheadings not included** | **Synonyms (free text)** |
| --- | --- | --- | --- |
| Appendicitis |  |  |  |
| Appendectomy |  |  | Appendix removal |
| Diverticular disease | Diverticulitis |  | Esophageal diverticulitis |
|  | Diverticulitis, esophageal |  | Oesophageal diverticulitis |
|  | Diverticulitis, colonic |  | Colonic diverticulitis |
|  | Diverticulitis, stomach |  | Gastric diverticulitis |
|  | Diverticulum |  | Esophageal diverticulum |
|  | Diverticulum, esophageal |  | Oesophageal diverticulum |
|  | Diverticulum, colonic |  | Colonic diverticulum |
|  | Diverticulum, stomach |  | Gastric diverticulum |
|  | Diverticulum, Meckel |  | Meckel’s diverticulum |
|  |  |  | Diverticular |
|  |  |  | Diverticular disease |
| Cholecystectomy | Cholecystectomy, laparoscopic |  | Laparoscopic cholecystectomy |
|  |  |  | Gallbladder removal |
|  |  |  | Gallbladder resection |
| Cholecystostomy |  |  |  |
| Cholecystitis | Acalculous cholecystitis |  | Acute cholecystitis |
|  | Cholecystitis, acute |  | Gallbladder disease |
|  | Emphysematous cholecystitis |  | Gallstone disease |
|  |  |  | Biliary colic |
|  |  |  | Common bile duct stone disease |
|  |  |  | CBD stone disease |
| Pancreatitis | Pancreatitis, acute haemorrhagic | Pancreatitis, chronic | Acute pancreatitis |
|  | Pancreatitis, acute necrotizing | Pancreatitis, graft | Acute haemorrhagic pancreatitis |
|  | Pancreatitis, alcoholic |  | Acute hemorrhagic pancreatitis |
|  |  |  | Acute necrotizing pancreatitis |
|  |  |  | Acute necrotising pancreatitis |
|  |  |  | Gallstone pancreatitis |
|  |  |  | Alcoholic pancreatitis |
| Abscess | Abdominal abscess | Liver abscess | Incisional abscess |
|  | Subphrenic abscess | Brain abscess | Perianal abscess |
|  | Psoas abscess | Epidural abscess | Peri-anal abscess |
|  |  | Lung abscess | Anorectal abscess |
|  |  | Periapical abscess | Ano-rectal abscess |
|  |  | Periodontal abscess | Anal abscess |
|  |  | Peritonsillar abscess | Rectal abscess |
|  |  | Retropharyngeal abscess |  |
| Hernia | Hernia, abdominal | Encephalocele | Abdominal hernia |
|  | Hernia, femoral | Gastroschisis | Femoral hernia |
|  | Hernia, ventral | Intervertebral disc displacement | Ventral hernia |
|  | Hernia, umbilical | Meningocele | Umbilical hernia |
|  | Hernia, inguinal |  | Inguinal hernia |
|  | Hernia, obturator |  | Obturator hernia |
|  | Incisional hernia |  | Diaphragmatic hernia |
|  | Internal hernia |  | Hiatus hernia |
|  | Hernia, diaphragmatic |  | Hiatal hernia |
|  | Hernia, diaphragmatic, congenital |  |  |
|  | Hernia, diaphragmatic, traumatic |  |  |
|  | Hernia, hiatal |  |  |
|  | Paraduodenal hernia |  |  |
|  |  |  | EGS |
|  |  |  | Emergency general surgery |
|  |  |  | Emergency surgery |
|  |  |  | Emergency surgical procedure |

**Specific Search Strategy for Databases including Filters and Limits Used:**

1 Ambulatory Care/ 47935

2 Ambulatory Surgical Procedures/ 13906

3 Outpatients/ 23743

4 outpatient.mp. [mp=title, book title, abstract, original title, name of substance word, subject heading word, floating sub-heading word, keyword heading word, organism supplementary concept word, protocol supplementary concept word, rare disease supplementary concept word, unique identifier, synonyms, population supplementary concept word, anatomy supplementary concept word] 199780

5 out-patient.mp. [mp=title, book title, abstract, original title, name of substance word, subject heading word, floating sub-heading word, keyword heading word, organism supplementary concept word, protocol supplementary concept word, rare disease supplementary concept word, unique identifier, synonyms, population supplementary concept word, anatomy supplementary concept word] 12290

6 out-patients.mp. [mp=title, book title, abstract, original title, name of substance word, subject heading word, floating sub-heading word, keyword heading word, organism supplementary concept word, protocol supplementary concept word, rare disease supplementary concept word, unique identifier, synonyms, population supplementary concept word, anatomy supplementary concept word] 6813

7 Day Care, Medical/ 5325

8 day care.mp. [mp=title, book title, abstract, original title, name of substance word, subject heading word, floating sub-heading word, keyword heading word, organism supplementary concept word, protocol supplementary concept word, rare disease supplementary concept word, unique identifier, synonyms, population supplementary concept word, anatomy supplementary concept word] 16065

9 day case.mp. [mp=title, book title, abstract, original title, name of substance word, subject heading word, floating sub-heading word, keyword heading word, organism supplementary concept word, protocol supplementary concept word, rare disease supplementary concept word, unique identifier, synonyms, population supplementary concept word, anatomy supplementary concept word] 3861

10 hot clinic.mp. [mp=title, book title, abstract, original title, name of substance word, subject heading word, floating sub-heading word, keyword heading word, organism supplementary concept word, protocol supplementary concept word, rare disease supplementary concept word, unique identifier, synonyms, population supplementary concept word, anatomy supplementary concept word] 11

11 office based.mp. [mp=title, book title, abstract, original title, name of substance word, subject heading word, floating sub-heading word, keyword heading word, organism supplementary concept word, protocol supplementary concept word, rare disease supplementary concept word, unique identifier, synonyms, population supplementary concept word, anatomy supplementary concept word] 6351

12 office-based.mp. [mp=title, book title, abstract, original title, name of substance word, subject heading word, floating sub-heading word, keyword heading word, organism supplementary concept word, protocol supplementary concept word, rare disease supplementary concept word, unique identifier, synonyms, population supplementary concept word, anatomy supplementary concept word] 6351

13 1 or 2 or 3 or 4 or 5 or 6 or 7 or 8 or 9 or 10 or 11 or 12 281636

14 Appendicitis/ 22158

15 Appendectomy/ 13387

16 appendix removal.mp. [mp=title, book title, abstract, original title, name of substance word, subject heading word, floating sub-heading word, keyword heading word, organism supplementary concept word, protocol supplementary concept word, rare disease supplementary concept word, unique identifier, synonyms, population supplementary concept word, anatomy supplementary concept word] 19

17 diverticular diseases/ or diverticulitis/ or diverticulitis, colonic/ or diverticulosis, esophageal/ or diverticulosis, stomach/ or diverticulum/ or diverticulum, colon/ or diverticulum, esophageal/ or diverticulum, stomach/ or meckel diverticulum/ 24388

18 Esophageal diverticulitis.mp. [mp=title, book title, abstract, original title, name of substance word, subject heading word, floating sub-heading word, keyword heading word, organism supplementary concept word, protocol supplementary concept word, rare disease supplementary concept word, unique identifier, synonyms, population supplementary concept word, anatomy supplementary concept word] 5

19 Oesophageal diverticulitis.mp. [mp=title, book title, abstract, original title, name of substance word, subject heading word, floating sub-heading word, keyword heading word, organism supplementary concept word, protocol supplementary concept word, rare disease supplementary concept word, unique identifier, synonyms, population supplementary concept word, anatomy supplementary concept word] 1

20 Colonic diverticulitis.mp. [mp=title, book title, abstract, original title, name of substance word, subject heading word, floating sub-heading word, keyword heading word, organism supplementary concept word, protocol supplementary concept word, rare disease supplementary concept word, unique identifier, synonyms, population supplementary concept word, anatomy supplementary concept word] 739

21 Gastric diverticulitis.mp. [mp=title, book title, abstract, original title, name of substance word, subject heading word, floating sub-heading word, keyword heading word, organism supplementary concept word, protocol supplementary concept word, rare disease supplementary concept word, unique identifier, synonyms, population supplementary concept word, anatomy supplementary concept word] 2

22 Esophageal diverticulum.mp. [mp=title, book title, abstract, original title, name of substance word, subject heading word, floating sub-heading word, keyword heading word, organism supplementary concept word, protocol supplementary concept word, rare disease supplementary concept word, unique identifier, synonyms, population supplementary concept word, anatomy supplementary concept word] 543

23 Oesophageal diverticulum.mp. [mp=title, book title, abstract, original title, name of substance word, subject heading word, floating sub-heading word, keyword heading word, organism supplementary concept word, protocol supplementary concept word, rare disease supplementary concept word, unique identifier, synonyms, population supplementary concept word, anatomy supplementary concept word] 90

24 Colonic diverticulum.mp. [mp=title, book title, abstract, original title, name of substance word, subject heading word, floating sub-heading word, keyword heading word, organism supplementary concept word, protocol supplementary concept word, rare disease supplementary concept word, unique identifier, synonyms, population supplementary concept word, anatomy supplementary concept word] 247

25 Gastric diverticulum.mp. [mp=title, book title, abstract, original title, name of substance word, subject heading word, floating sub-heading word, keyword heading word, organism supplementary concept word, protocol supplementary concept word, rare disease supplementary concept word, unique identifier, synonyms, population supplementary concept word, anatomy supplementary concept word] 177

26 Meckel's diverticulum.mp. [mp=title, book title, abstract, original title, name of substance word, subject heading word, floating sub-heading word, keyword heading word, organism supplementary concept word, protocol supplementary concept word, rare disease supplementary concept word, unique identifier, synonyms, population supplementary concept word, anatomy supplementary concept word] 4028

27 Diverticular.mp. [mp=title, book title, abstract, original title, name of substance word, subject heading word, floating sub-heading word, keyword heading word, organism supplementary concept word, protocol supplementary concept word, rare disease supplementary concept word, unique identifier, synonyms, population supplementary concept word, anatomy supplementary concept word] 5774

28 Diverticular disease.mp. [mp=title, book title, abstract, original title, name of substance word, subject heading word, floating sub-heading word, keyword heading word, organism supplementary concept word, protocol supplementary concept word, rare disease supplementary concept word, unique identifier, synonyms, population supplementary concept word, anatomy supplementary concept word] 3802

29 cholecystectomy/ or cholecystectomy, laparoscopic/ 32169

30 Laparoscopic cholecystectomy.mp. [mp=title, book title, abstract, original title, name of substance word, subject heading word, floating sub-heading word, keyword heading word, organism supplementary concept word, protocol supplementary concept word, rare disease supplementary concept word, unique identifier, synonyms, population supplementary concept word, anatomy supplementary concept word] 16757

31 Gallbladder removal.mp. [mp=title, book title, abstract, original title, name of substance word, subject heading word, floating sub-heading word, keyword heading word, organism supplementary concept word, protocol supplementary concept word, rare disease supplementary concept word, unique identifier, synonyms, population supplementary concept word, anatomy supplementary concept word] 192

32 Gallbladder resection.mp. [mp=title, book title, abstract, original title, name of substance word, subject heading word, floating sub-heading word, keyword heading word, organism supplementary concept word, protocol supplementary concept word, rare disease supplementary concept word, unique identifier, synonyms, population supplementary concept word, anatomy supplementary concept word] 31

33 Cholecystostomy/ 970

34 cholecystitis/ or acalculous cholecystitis/ or cholecystitis, acute/ or emphysematous cholecystitis/ 16090

35 Acute cholecystitis.mp. [mp=title, book title, abstract, original title, name of substance word, subject heading word, floating sub-heading word, keyword heading word, organism supplementary concept word, protocol supplementary concept word, rare disease supplementary concept word, unique identifier, synonyms, population supplementary concept word, anatomy supplementary concept word] 8139

36 Gallbladder disease.mp. [mp=title, book title, abstract, original title, name of substance word, subject heading word, floating sub-heading word, keyword heading word, organism supplementary concept word, protocol supplementary concept word, rare disease supplementary concept word, unique identifier, synonyms, population supplementary concept word, anatomy supplementary concept word] 2101

37 Gallstone disease.mp. [mp=title, book title, abstract, original title, name of substance word, subject heading word, floating sub-heading word, keyword heading word, organism supplementary concept word, protocol supplementary concept word, rare disease supplementary concept word, unique identifier, synonyms, population supplementary concept word, anatomy supplementary concept word] 3407

38 Biliary colic.mp. [mp=title, book title, abstract, original title, name of substance word, subject heading word, floating sub-heading word, keyword heading word, organism supplementary concept word, protocol supplementary concept word, rare disease supplementary concept word, unique identifier, synonyms, population supplementary concept word, anatomy supplementary concept word] 1121

39 common bile duct stone disease.mp. [mp=title, book title, abstract, original title, name of substance word, subject heading word, floating sub-heading word, keyword heading word, organism supplementary concept word, protocol supplementary concept word, rare disease supplementary concept word, unique identifier, synonyms, population supplementary concept word, anatomy supplementary concept word] 6

40 CBD stone disease.mp. [mp=title, book title, abstract, original title, name of substance word, subject heading word, floating sub-heading word, keyword heading word, organism supplementary concept word, protocol supplementary concept word, rare disease supplementary concept word, unique identifier, synonyms, population supplementary concept word, anatomy supplementary concept word] 3

41 pancreatitis/ or pancreatitis, acute hemorrhagic/ or pancreatitis, acute necrotizing/ or pancreatitis, alcoholic/ 54165

42 Acute pancreatitis.mp. [mp=title, book title, abstract, original title, name of substance word, subject heading word, floating sub-heading word, keyword heading word, organism supplementary concept word, protocol supplementary concept word, rare disease supplementary concept word, unique identifier, synonyms, population supplementary concept word, anatomy supplementary concept word] 30612

43 Acute haemorrhagic pancreatitis.mp. [mp=title, book title, abstract, original title, name of substance word, subject heading word, floating sub-heading word, keyword heading word, organism supplementary concept word, protocol supplementary concept word, rare disease supplementary concept word, unique identifier, synonyms, population supplementary concept word, anatomy supplementary concept word] 91

44 Acute hemorrhagic pancreatitis.mp. [mp=title, book title, abstract, original title, name of substance word, subject heading word, floating sub-heading word, keyword heading word, organism supplementary concept word, protocol supplementary concept word, rare disease supplementary concept word, unique identifier, synonyms, population supplementary concept word, anatomy supplementary concept word] 376

45 Acute necrotizing pancreatitis.mp. [mp=title, book title, abstract, original title, name of substance word, subject heading word, floating sub-heading word, keyword heading word, organism supplementary concept word, protocol supplementary concept word, rare disease supplementary concept word, unique identifier, synonyms, population supplementary concept word, anatomy supplementary concept word] 1330

46 Acute necrotising pancreatitis.mp. [mp=title, book title, abstract, original title, name of substance word, subject heading word, floating sub-heading word, keyword heading word, organism supplementary concept word, protocol supplementary concept word, rare disease supplementary concept word, unique identifier, synonyms, population supplementary concept word, anatomy supplementary concept word] 129

47 Gallstone pancreatitis.mp. [mp=title, book title, abstract, original title, name of substance word, subject heading word, floating sub-heading word, keyword heading word, organism supplementary concept word, protocol supplementary concept word, rare disease supplementary concept word, unique identifier, synonyms, population supplementary concept word, anatomy supplementary concept word] 914

48 Alcoholic pancreatitis.mp. [mp=title, book title, abstract, original title, name of substance word, subject heading word, floating sub-heading word, keyword heading word, organism supplementary concept word, protocol supplementary concept word, rare disease supplementary concept word, unique identifier, synonyms, population supplementary concept word, anatomy supplementary concept word] 1028

49 abscess/ or abdominal abscess/ or subphrenic abscess/ or psoas abscess/ 35014

50 Incisional abscess.mp. [mp=title, book title, abstract, original title, name of substance word, subject heading word, floating sub-heading word, keyword heading word, organism supplementary concept word, protocol supplementary concept word, rare disease supplementary concept word, unique identifier, synonyms, population supplementary concept word, anatomy supplementary concept word] 5

51 Perianal abscess.mp. [mp=title, book title, abstract, original title, name of substance word, subject heading word, floating sub-heading word, keyword heading word, organism supplementary concept word, protocol supplementary concept word, rare disease supplementary concept word, unique identifier, synonyms, population supplementary concept word, anatomy supplementary concept word] 760

52 Peri-anal abscess.mp. [mp=title, book title, abstract, original title, name of substance word, subject heading word, floating sub-heading word, keyword heading word, organism supplementary concept word, protocol supplementary concept word, rare disease supplementary concept word, unique identifier, synonyms, population supplementary concept word, anatomy supplementary concept word] 23

53 Anorectal abscess.mp. [mp=title, book title, abstract, original title, name of substance word, subject heading word, floating sub-heading word, keyword heading word, organism supplementary concept word, protocol supplementary concept word, rare disease supplementary concept word, unique identifier, synonyms, population supplementary concept word, anatomy supplementary concept word] 151

54 Ano-rectal abscess.mp. [mp=title, book title, abstract, original title, name of substance word, subject heading word, floating sub-heading word, keyword heading word, organism supplementary concept word, protocol supplementary concept word, rare disease supplementary concept word, unique identifier, synonyms, population supplementary concept word, anatomy supplementary concept word] 5

55 Anal abscess.mp. [mp=title, book title, abstract, original title, name of substance word, subject heading word, floating sub-heading word, keyword heading word, organism supplementary concept word, protocol supplementary concept word, rare disease supplementary concept word, unique identifier, synonyms, population supplementary concept word, anatomy supplementary concept word] 140

56 Rectal abscess.mp. [mp=title, book title, abstract, original title, name of substance word, subject heading word, floating sub-heading word, keyword heading word, organism supplementary concept word, protocol supplementary concept word, rare disease supplementary concept word, unique identifier, synonyms, population supplementary concept word, anatomy supplementary concept word] 74

57 hernia/ or hernia, abdominal/ or hernia, femoral/ or hernia, inguinal/ or hernia, ventral/ or hernia, umbilical/ or hernia, obturator/ or incisional hernia/ or internal hernia/ or hernia, diaphragmatic/ or hernias, diaphragmatic, congenital/ or hernia, diaphragmatic, traumatic/ or hernia, hiatal/ or paraduodenal hernia/ 59289

58 Abdominal hernia.mp. [mp=title, book title, abstract, original title, name of substance word, subject heading word, floating sub-heading word, keyword heading word, organism supplementary concept word, protocol supplementary concept word, rare disease supplementary concept word, unique identifier, synonyms, population supplementary concept word, anatomy supplementary concept word] 1023

59 Femoral hernia.mp. [mp=title, book title, abstract, original title, name of substance word, subject heading word, floating sub-heading word, keyword heading word, organism supplementary concept word, protocol supplementary concept word, rare disease supplementary concept word, unique identifier, synonyms, population supplementary concept word, anatomy supplementary concept word] 1301

60 Ventral hernia.mp. [mp=title, book title, abstract, original title, name of substance word, subject heading word, floating sub-heading word, keyword heading word, organism supplementary concept word, protocol supplementary concept word, rare disease supplementary concept word, unique identifier, synonyms, population supplementary concept word, anatomy supplementary concept word] 3966

61 Umbilical hernia.mp. [mp=title, book title, abstract, original title, name of substance word, subject heading word, floating sub-heading word, keyword heading word, organism supplementary concept word, protocol supplementary concept word, rare disease supplementary concept word, unique identifier, synonyms, population supplementary concept word, anatomy supplementary concept word] 2279

62 Inguinal hernia.mp. [mp=title, book title, abstract, original title, name of substance word, subject heading word, floating sub-heading word, keyword heading word, organism supplementary concept word, protocol supplementary concept word, rare disease supplementary concept word, unique identifier, synonyms, population supplementary concept word, anatomy supplementary concept word] 14141

63 Obturator hernia.mp. [mp=title, book title, abstract, original title, name of substance word, subject heading word, floating sub-heading word, keyword heading word, organism supplementary concept word, protocol supplementary concept word, rare disease supplementary concept word, unique identifier, synonyms, population supplementary concept word, anatomy supplementary concept word] 606

64 Diaphragmatic hernia.mp. [mp=title, book title, abstract, original title, name of substance word, subject heading word, floating sub-heading word, keyword heading word, organism supplementary concept word, protocol supplementary concept word, rare disease supplementary concept word, unique identifier, synonyms, population supplementary concept word, anatomy supplementary concept word] 11238

65 Hiatus hernia.mp. [mp=title, book title, abstract, original title, name of substance word, subject heading word, floating sub-heading word, keyword heading word, organism supplementary concept word, protocol supplementary concept word, rare disease supplementary concept word, unique identifier, synonyms, population supplementary concept word, anatomy supplementary concept word] 2198

66 Hiatal hernia.mp. [mp=title, book title, abstract, original title, name of substance word, subject heading word, floating sub-heading word, keyword heading word, organism supplementary concept word, protocol supplementary concept word, rare disease supplementary concept word, unique identifier, synonyms, population supplementary concept word, anatomy supplementary concept word] 5386

67 EGS.mp. [mp=title, book title, abstract, original title, name of substance word, subject heading word, floating sub-heading word, keyword heading word, organism supplementary concept word, protocol supplementary concept word, rare disease supplementary concept word, unique identifier, synonyms, population supplementary concept word, anatomy supplementary concept word] 1444

68 Emergency general surgery.mp. [mp=title, book title, abstract, original title, name of substance word, subject heading word, floating sub-heading word, keyword heading word, organism supplementary concept word, protocol supplementary concept word, rare disease supplementary concept word, unique identifier, synonyms, population supplementary concept word, anatomy supplementary concept word] 1154

69 Emergency surgery.mp. [mp=title, book title, abstract, original title, name of substance word, subject heading word, floating sub-heading word, keyword heading word, organism supplementary concept word, protocol supplementary concept word, rare disease supplementary concept word, unique identifier, synonyms, population supplementary concept word, anatomy supplementary concept word] 12833

70 Emergency surgical procedure.mp. [mp=title, book title, abstract, original title, name of substance word, subject heading word, floating sub-heading word, keyword heading word, organism supplementary concept word, protocol supplementary concept word, rare disease supplementary concept word, unique identifier, synonyms, population supplementary concept word, anatomy supplementary concept word] 117

71 14 or 15 or 16 or 17 or 18 or 19 or 20 or 21 or 22 or 23 or 24 or 25 or 26 or 27 or 28 or 29 or 30 or 31 or 32 or 33 or 34 or 35 or 36 or 37 or 38 or 39 or 40 or 41 or 42 or 43 or 44 or 45 or 46 or 47 or 48 or 49 or 50 or 51 or 52 or 53 or 54 or 55 or 56 or 57 or 58 or 59 or 60 or 61 or 62 or 63 or 64 or 65 or 66 or 67 or 68 or 69 or 70278023

72 13 and 71 3425

73 72 3425

74 limit 73 to (english language and full text) 549

75 74 549

76 limit 75 to yr="Dec 2018 – Dec 2023" 93

**Appendix 2 – PROSPERO Registration Information**

**PROSPERO**

International prospective register of systematic reviews

**Current evidence for the ambulatory management of emergency general surgical conditions: a systematic review**

Review methods were amended after registration. Please see the revision notes and previous versions for detail.

## Citation

PROSPERO 2024 Available from <https://www.crd.york.ac.uk/PROSPERO/view/CRD42023468866>

# REVIEW TITLE AND BASIC DETAILS

## Review title

Current evidence for the ambulatory management of emergency general surgical conditions: a systematic review

## Review objectives

Is there any evidence of the effects of ambulatory pathways in emergency general surgery on clinical outcomes (admission/re-admission rate, recovery time, patient satisfaction, complications, mortality) and cost?
If so, what is the quality of this evidence and what does it show?

## Keywords

Abscess, Acute cholecystitis, Acute pancreatitis, Ambulatory care, Appendicitis, Biliary disease, Diverticular disease, Efficacy, Emergency general surgery, Hernia, Safety, Systematic review

# SEARCHING AND SCREENING

## Searches 1 change

We will search the following electronic databases: EMBASE, MEDLINE OVID, PubMed, Cochrane Register. Searches will be restricted to English language, full-text articles only and looking at any studies published within the last 5 years.

## Study design 1 change

Inclusion: randomised controlled trials, case-control studies, cohort studies
Exclusion: editorials, expert opinion articles, case reports, conference proceedings, letters, case series
This is to ensure that only higher levels of evidence are included, while keeping the scope of the search broad enough to ensure an adequate quantity of evidence is collected for a comprehensive review.

# ELIGIBILITY CRITERIA

## Condition or domain being studied

Ambulatory care in emergency general surgery
Conditions included: appendicitis, acute cholecystitis, biliary disease, diverticulitis, abdominal or digestive tract abscesses, abdominal hernias, acute pancreatitis

## Population 1 change

Inclusion: patients undergoing emergency general surgical procedures, patients under the care of the emergency general surgery team or who have presented with a condition normally covered by this team (appendicitis, cholecystitis, diverticulitis, abdominal or digestive tract abscesses, abdominal hernias, acute pancreatitis)
Exclusion: animal studies, patients with chronic surgical conditions as the primary focus, paediatric patients, non-surgical patients

## Intervention(s) or exposure(s) 1 change

Ambulatory care is defined as medical care provided on an outpatient basis. This may include care provided in a daily or weekly clinic, or day surgery where patients are not admitted for overnight stay.
Inclusion: any patient under the care of the emergency general surgery team or with a condition typically covered by this team, who is being cared for in an ambulatory care setting, studies performed in a recognised secondary care setting
Exclusion: patients being cared for in a primary care setting, patients whose care is primarily focused on a non-surgical condition, single post-operative follow-up visits in the absence of a wider ambulatory pathway

## Comparator(s) or control(s)

Inpatient care is defined as any patient who is admitted to hospital for overnight stay.
Inclusion: any patient under the care of the emergency general surgery team or with a condition typically covered by this team, who is being cared for in an inpatient setting
Some studies may not have comparators but will also be included in the review.

## Context

# OUTCOMES TO BE ANALYSED

## Main outcomes 1 change

The primary outcome of this review is to summarise the current evidence base on ambulatory care in emergency general surgery. To do this, we will examine ambulatory protocols used and explore clinical, quality of life and health economic outcomes.

### *Measures of effect*

Measures of clinical outcome: rates of admission and re-admission, rates of complications, and mortality rates

## Additional outcomes 1 change

Measures of patient satisfaction: any quantitative measure using a validated patient satisfaction tool
Measures of cost: total/mean hospital expenses, percentage cost savings

# DATA COLLECTION PROCESS

## Data extraction (selection and coding) 1 change

Results from the search will be directly exported into an Excel reference list from which results will be filtered to remove duplicates and non-research papers (letters, editorials, conference proceedings). Remaining results will be uploaded to Rayyan software for screening. Titles and abstracts will be screened independently by two reviewers. Full texts of these potentially eligible studies will be retrieved and assessed independently for eligibility by two reviewers. Any disagreements during these stages will be primarily resolved by discussion between the two reviewers, and a third reviewer’s opinion sought if disagreement persists.
Data extraction will be performed using a form developed during the protocol phase. For each included study, data will be collected on author, year of publication, journal of publication, study design, sample size, location of the trial, participating centres, the condition(s) studied, the methods of ambulatory management used, and any outcomes of interest. Data will be extracted by one reviewer, and checked by another reviewer. Any disagreements will be discussed and a third reviewer will be involved where agreement cannot be reached. Where necessary, study authors will be contacted to provide further information.

## Risk of bias (quality) assessment

We will use the Cochrane tool RoB-2 for assessing risk of bias in randomised trials, and the Cochrane tool ROBINS-I for assessing risk of bias in non-randomised studies of the effects of interventions (including case-control studies and cohort studies). Eligible studies will be assessed independently for risk of bias by two reviewers. Disagreements will be discussed between the two reviewers, with the involvement of a third reviewer where necessary.

# PLANNED DATA SYNTHESIS

## Strategy for data synthesis

Ambulatory protocols for a range of general surgical conditions will be examined. Studies will be grouped firstly according to disease group (such as pancreatitis or appendicitis), and then further subgrouping will be performed based on the outcome studied, for example cost or patient outcomes. A narrative summary of the results is planned. Study quality will be explored using the appropriate risk of bias tools discussed above - RoB-2 for randomised trials and ROBINS-I for non-randomised studies. As we are expecting significant heterogeneity in the disease/condition studied, the method of intervention (i.e the ambulatory protocol used) and the primary outcome(s) of each study, no formal meta-analysis will be undertaken.

## Analysis of subgroups or subsets

As we anticipate a significant heterogeneity in the conditions of interest, interventions, and primary outcome(s) of the studies, we will primarily group studies by disease or condition of interest, as discussed above. Where these groups contain multiple studies, we will then perform subgrouping according to primary outcome to perform data synthesis as described above.

# REVIEW AFFILIATION, FUNDING AND PEER REVIEW

## Review affiliation

Leeds Institute of Emergency General Surgery, Leeds Teaching Hospitals Trust, NHS

Leeds Pancreas Unit, Leeds Teaching Hospitals Trust, NHS

## Funding source

Surgical Research Award Grant received from Association of Surgeons of Great Britain and Northern Ireland in association with GUTS UK

## Named contact

Mary Walters.
walters.mary@hotmail.co.uk

# TIMELINE OF THE REVIEW

## Review timeline

Start date: 28 August 2023. End date: 15 April 2024

## Date of first submission to PROSPERO

02 October 2023

## Date of registration in PROSPERO

06 November 2023

# CURRENT REVIEW STAGE

## Publication of review results

The intention is to publish the review once completed.The review will be published in English

## Review status

The review is currently planned or ongoing.

# ADDITIONAL INFORMATION

## PROSPERO version history

- Version 1.2 published on 18 Mar 2024
- Version 1.1 published on 15 Mar 2024
- Version 1.0 published on 06 Nov 2023

## Review conflict of interest

None known

## Country

England

## Medical Subject Headings

Acute Care Surgery; Hospital Mortality; Humans; Patient Satisfaction; Postoperative Complications

## Revision note 1 change

Changes to inclusion criteria: Paediatric studies excluded (to allow focus purely on adult ambulatory surgical pathways, as process of ambulation between largely independent adults and dependent children varies significantly). Types of study excluded updated to include case series as level 5 evidence (and therefore not eligible for inclusion).Addition of exclusion criterion of studies describing single post-operative follow-up visits with no further ambulatory pathway - ambulatory pathways not well-defined in the literature and the term was used to describe a heterogeneous group of studies - change made to attempt to limit results to only those fitting our prior definitions of ambulatory pathway. Changes to study selection protocol - described use of Excel for initial filtering of results, then uploading results to Rayyan for screening. Change made to reflect actual process of screening (using best software available and known to us at time of review). Updated databases searched (changes made from original protocol based on medical librarian's access to different databases)

## Disclaimer

The content of this record displays the information provided by the review team. PROSPERO does not peer review registration records or endorse their content.

PROSPERO accepts and posts the information provided in good faith; responsibility for record content rests with the review team. The owner of this record has affirmed that the information provided is truthful and that they understand that deliberate provision of inaccurate information may be construed as scientific misconduct.

PROSPERO does not accept any liability for the content provided in this record or for its use. Readers use the information provided in this record at their own risk.

| **Appendix 3 – Demographics of Included Studies** | | | | | | | | | |
| --- | --- | --- | --- | --- | --- | --- | --- | --- | --- |
| ***No:*** | ***Author:*** | ***Journal:*** | ***Year:*** | ***Country:*** | ***Type of Study:*** | ***Disease process(es):*** | ***Centres (n):*** | ***Patients (n):*** | ***Patients on ambulatory pathway (n/%)*** |
| *1* | *Pecere et al* | *Eur Review Med Pharmacol Sci* | *2020* | *Italy* | *Retrospective cohort study* | *Diverticular disease* | *1* | *166* | *58 (35)* |
| *2* | *Hickland et al* | *Ann R Coll Surg Engl* | *2021* | *UK* | *Prospective and retrospective cohort study* | *23 separate disease processes inc gen surg, urology, gynaecology* | *1* | *564* | *69 (12)* |
| *3* | *Raimbert et al* | *Surgery* | *2023* | *France* | *Prospective cohort study* | *Appendicitis* | *1* | *1730* | *451 (26)* |
| *4* | *Hajri et al* | *Pan Afr Med J* | *2022* | *Tunisia* | *Retrospective cohort study* | *Groin/ventral hernia* | *1* | *1294* | *1294 (100)* |
| *5* | *Writing Group for the CODA Collaborative* | *JAMA Netw Open* | *2022* | *USA* | *Retrospective cohort study* | *Appendicitis* | *25* | *726* | *335 (46.1)* |
| *6* | *Ciyiltepe et al* | *Ulus Travma Acil Cerrahi Derg* | *2021* | *Turkey* | *Prospective cohort study* | *Biliary disease* | *1* | *72* | *36 (50)* |
| *7* | *Edison et al* | *BMJ Open Qual* | *2021* | *UK* | *Prospective cohort study* | *Biliary disease, abscesses, groin hernias* | *1* | *175* | *80 (45.7)* |
| *8* | *Demetrashvili et al* | *Ann Med Surg* | *2019* | *Georgia* | *Prospective cohort study* | *Appendiceal mass/abscess* | *1* | *74* | *23 (31.1)* |
| *9* | *Enodien et al* | *Int J Environ Res Public Health* | *2022* | *Switzerland* | *Retrospective cohort study* | *Inguinal hernia* | *1* | *234* | *146 (62.4)* |
| *10* | *Cullen et al* | *Surg Endosc* | *2021* | *Tanzania* | *Mixed methods (retrospective cohort and qualitative) study* | *Biliary disease* | *1* | *147* | *109 (74.1)*  *Successful ambulation 82 (55.8)* |
| *11* | *Joyner et al* | *Hernia* | *2023* | *UK* | *Retrospective cohort study* | *Inguinal hernia* | *161* | *413,059* | *326,833 (79.1)* |
| *12* | *Pham et al* | *ANZ J Surg* | *2021* | *Australia* | *Retrospective cohort study* | *Biliary disease* | *1* | *282* | *169 (59.9)*  *Successful ambulation:*  *124 (44.0)* |
| *13* | *Janeway et al* | *J Surg Res* | *2021* | *USA* | *Retrospective cohort study* | *Biliary disease* | *Unknown (data from 3 US states)* | *321,335* | *190,734 (59.4)* |
| *14* | *Trejo-Avila et al* | *Surg Endosc* | *2019* | *Mexico* | *Randomised controlled trial* | *Appendicitis* | *1* | *108* | *50 (46.3)* |
| *15* | *Abet et al* | *J Visc Surg* | *2023* | *France* | *Prospective cohort study* | *Diverticular disease* | *1* | *87* | *59 (67.8)* |
| *16* | *Sala-Hernandez et al* | *Cirugia Espanola* | *2019* | *Spain* | *Retrospective cohort study* | *Biliary disease, pancreatitis* | *1* | *260* | *260 (100)* |
| *17* | *Subirana Magdaleno et al* | *Cirugia Espanola* | *2018* | *Spain* | *Prospective randomised controlled trial* | *Biliary disease* | *1* | *62* | *62 (100)* |
| *18* | *Drissi et al* | *J Visc Surg* | *2019* | *France* | *Prospective cohort study* | *Incisional hernia* | *Unknown (nationwide database)* | *1429* | *305 (21.3)*  *Successful ambulation: 272 (19)* |
| *19* | *Gaszynski et al* | *ANZ J Surg* | *2019* | *Australia* | *Retrospective cohort study* | *Superficial skin abscess* | *1* | *100* | *50 (50)* |
| *20* | *Qu et al* | *HBPD Int* | *2019* | *China* | *Randomised controlled trial* | *Biliary disease* | *1* | *91* | *91 (100)* |
| *21* | *Rosero et al* | *Acta Anaesthesiol Scanda* | *2019* | *USA* | *Retrospective cohort study* | *Hernia* | *Unknown (national database)* | *214,125* | *214,125 (100)* |
| *22* | *Riche et al* | *J Gynecol Obstet Hum Reprod* | *2020* | *France* | *Retrospective cohort study* | *Bartholin’s abscess* | *Unknown (national database)* | *3539* | *0 (0)* |
| *23* | *Alvarez-Aguilera et al* | *Hernia* | *2023* | *Spain* | *Retrospective cohort study* | *Groin hernia* | *1* | *743* | *344 (46.2)* |
| *24* | *Mora-Lopez et al* | *Ann Surg* | *2021* | *Spain* | *Randomised controlled trial* | *Diverticular disease* | *15* | *480* | *480 (100)* |
| *25* | *Teke et al* | *Med Bull Sisli Etfal Hosp* | *2022* | *Turkey* | *Retrospective cohort study* | *Diverticular disease* | *1* | *172* | *62 (36)* |
| *26* | *Sabbagh et al* | *JACS* | *2019* | *France* | *Retrospective cohort study* | *Appendicitis* | *1* | *296* | *201 (67.9)* |
| *27* | *Taylor Martin et al* | *Am J Surg* | *2023* | *USA* | *Retrospective cohort study* | *Biliary disease* | *1* | *607* | *607 (100)* |
| *28* | *McClintock et al* | *ANZ J Surg* | *2023* | *Australia* | *Randomised non-inferiority control trial* | *Diverticular disease* | *1* | *118* | *118 (100)* |
| *29* | *Pizza et al* | *Obes Surg* | *2021* | *Italy* | *Retrospective cohort study* | *Bariatric surgery* | *1* | *452* | *393 (86.9)* |
| *30* | *Guillaumes et al* | *Updates Surg* | *2023* | *Spain* | *Retrospective cohort study* | *Direct and indirect inguinal hernias* | *791* | *1,163,039* | *510,440 (43.9)* |
| *31* | *Elvira Lopez et al* | *World J Emerg Surg* | *2022* | *Spain* | *Randomised controlled trial* | *Appendicitis* | *1* | *97* | *49 (50.5)* |
| *32* | *Ceresoli et al* | *World J Surg* | *2023* | *Italy* | *Retrospective non-inferiority study* | *Appendicitis* | *4* | *668* | *147 (22)* |
| *33* | *Adler et al* | *Endosc Ultrasound* | *2019* | *USA* | *Multicentre retrospective cohort study* | *Pancreatic Pseudocysts/Walled off pancreatic necrosis* | *4* | *80* | *33 (41.3)* |
| *34* | *Massoumi et al* | *Am Surg* | *2020* | *USA* | *Retrospective cohort study* | *Anorectal surgery, biliary disease, groin and abdominal wall hernia* | *14* | *52,236* | *276 (0.53)* |
| *35* | *Juszczyk et al* | *ANZ J Surg* | *2019* | *Australia* | *Retrospective cohort study* | *Diverticular disease* | *1* | *1147* | *208 (18.1)* |
| *36* | *Unal et al* | *Turk Geriatri Derg* | *2018* | *Turkey* | *Retrospective cohort study* | *Direct and indirect inguinal hernias* | *1* | *370* | *368 (99.5)* |
| *37* | *Munoz-Cruzado et al* | *Ann Surg* | *2023* | *Spain* | *Randomised controlled trial* | *Appendicitis* | *1* | *300* | *149 (49.7)* |
| *38* | *Subirana et al* | *JMAS* | *2020* | *Spain* | *Randomised controlled trial* | *Biliary disease* | *1* | *73* | *37 (50.7)* |
| *39* | *Brungger et al* | *Health Policy* | *2021* | *Switzerland* | *Retrospective cross-sectional study* | *Haemorrhoidectomy, inguinal hernia repair, varicose vein surgery, knee arthroscopy or meniscectomy, surgery of the cervix or uterus* | *Unknown (health insurance data)* | *38,475* | *16,999 (44.2)* |
| *40* | *Cargnelli et al* | *CJEM* | *2019* | *Canada* | *Retrospective cohort study* | *Abdominal pain* | *1* | *299* | *299 (100)* |
| *41* | *Kockerling et al* | *Hernia* | *2022* | *Germany* | *Retrospective cohort study* | *Direct and indirect inguinal hernias* | *737* | *342,072* | *50,524 (14.8)* |
| *42* | *Oliveira et al* | *Revista Cientifica da Ordem dos Medicos* | *2022* | *Portugal* | *Retrospective case-control study* | *Abdominal wall hernia* | *1* | *136* | *71 (52.2)* |
| 43 | *Friedlander et al* | *Ann Surg* | *2021* | *USA* | *Retrospective cohort study* | *Groin and abdominal wall herniae, thyroid goitres, biliary disease, appendicitis* | *620* | *73,724* | *64215 (87.1)* |

**Appendix 4 – Reporting of the Components of Ambulatory Pathways across Included Papers**

| **Paper** | **SDM** | **Scoring and classification systems** | **Investigations** | **Care Escalation criteria** | **Discharge criteria** | **Follow-up** |
| --- | --- | --- | --- | --- | --- | --- |
| *Pecere et al,* 2020 | **✓** | **✓** | **✓** | **X** | **X** | **X** |
| *Hickland et al,* 2021 | **✓** | **X** | **X** | **X** | **X** | **✓** |
| *Raimbert et al,* 2023 | **X** | **✓** | **✓** | **X** | **X** | **✓** |
| *Hajri et al*, 2022 | **✓** | **✓** | **✓** | **X** | **✓** | **X** |
| *Writing Group for the CODA Collaborative*, 2022 | **✓** | **✓** | **✓** | **X** | **✓** | **✓** |
| *Ciyiltepe et al*, 2021 | **X** | **✓** | **✓** | **X** | **✓** | **X** |
| *Edison et al*, 2021 | **✓** | **✓** | **X** | **X** | **X** | **✓** |
| *Demetrashvili et al*, 2019 | **X** | **X** | **✓** | **X** | **X** | **✓** |
| *Enodien et al*, 2022 | **X** | **X** | **X** | **X** | **X** | **✓** |
| *Cullen et al*, 2021 | **✓** | **X** | **X** | **X** | **X** | **X** |
| *Joyner et al*, 2023 | **X** | **✓** | **X** | **X** | **X** | **X** |
| *Pham et al*, 2021 | **✓** | **✓** | **✓** | **X** | **✓** | **✓** |
| *Janeway et al*, 2021 | **X** | **✓** | **X** | **X** | **X** | **X** |
| *Trejo-Avila et al*, 2019 | **✓** | **✓** | **✓** | **✓** | **✓** | **✓** |
| *Abet et al*, 2023 | **✓** | **✓** | **✓** | **✓** | **✓** | **✓** |
| *Sala-Hernandez et al*, 2019 | **✓** | **✓** | **✓** | **✓** | **X** | **✓** |
| *Subirana Magdaleno et al*, 2018 | **✓** | **✓** | **✓** | **X** | **✓** | **✓** |
| *Drissi et al*, 2019 | **✓** | **✓** | **✓** | **X** | **✓** | **✓** |
| *Gaszynski et al*, 2019 | **✓** | **X** | **✓** | **X** | **X** | **✓** |
| *Qu et al*, 2019 | **X** | **✓** | **✓** | **✓** | **✓** | **✓** |
| *Rosero et al*, 2019 | **X** | **X** | **X** | **X** | **X** | **X** |
| *Riche et al*, 2020 | **X** | **X** | **✓** | **X** | **X** | **✓** |
| *Alvarez-Aguilera et al*, 2023 | **X** | **X** | **✓** | **X** | **X** | **X** |
| *Mora-Lopez et al*, 2021 | **X** | **✓** | **✓** | **✓** | **✓** | **✓** |
| *Teke et al*, 2022 | **✓** | **✓** | **✓** | **✓** | **✓** | **✓** |
| *Sabbagh et al*, 2019 | **✓** | **✓** | **✓** | **✓** | **✓** | **✓** |
| *Taylor Martin et al*, 2023 | **✓** | **X** | **✓** | **X** | **X** | **X** |
| *McClintock et al*, 2023 | **X** | **✓** | **✓** | **✓** | **✓** | **✓** |
| *Pizza et al*, 2021 | **X** | **X** | **✓** | **X** | **X** | **✓** |
| *Guillaumes et al*, 2023 | **X** | **X** | **✓** | **X** | **X** | **X** |
| *Elvira Lopez et al*, 2022 | **X** | **✓** | **✓** | **X** | **✓** | **✓** |
| *Ceresoli et al*, 2023 | **✓** | **✓** | **✓** | **X** | **X** | **✓** |
| *Adler et al*, 2019 | **✓** | **X** | **✓** | **X** | **X** | **✓** |
| *Massoumi et al*, 2020 | **X** | **X** | **✓** | **X** | **X** | **✓** |
| *Juszczyk et al*, 2019 | **✓** | **✓** | **✓** | **✓** | **✓** | **✓** |
| *Unal et al*, 2018 | **✓** | **✓** | **✓** | **X** | **✓** | **✓** |
| *Munoz-Cruzado et al*, 2023 | **✓** | **✓** | **X** | **X** | **✓** | **✓** |
| *Subirana et al*, 2020 | **X** | **✓** | **X** | **X** | **✓** | **✓** |
| *Brungger et al*, 2021 | **X** | **X** | **X** | **X** | **X** | **✓** |
| *Cargnelli et al*, 2019 | **✓** | **X** | **✓** | **X** | **X** | **✓** |
| *Kockerling et al*, 2022 | **✓** | **X** | **✓** | **X** | **X** | **X** |
| *Oliveira et al*, 2022 | **✓** | **X** | **X** | **X** | **X** | **X** |
| *Friedlander et al*, 2021 | **X** | **✓** | **✓** | **X** | **X** | **X** |
| Total N Reported (%) | **24 (56)** | **26 (60)** | **32 (74)** | **9 (21)** | **18 (42)** | **30 (70)** |

**Appendix 5 - Comparison of Matched Pathways**

| **Paper** | **SDM** | **Selection criteria** | **Discharge criteria** | **Readmission criteria** | **Scoring & classification systems** | **Follow-up** |
| --- | --- | --- | --- | --- | --- | --- |
| **Hernia** | | | | | | |
| 1 | “The Surgeon”, anaesthetsist | - | Stable vital signs, tolerating liquid diet, pain control, spontaneous micturition, no nausea and vomiting, patient agreement, presence of adult companion | - | Aldrete | - |
| 2 | - | - | - | - | - | Clinic |
| 3 | - | - | - | - | HFRS, IMD | - |
| 4 | Surgical consultant, anaesthetist | - | Stable vital signs, absence of bleeding or pain, oral intake, deambulation, urination | - | Clavien Dindo | Clinic |
| 5 | - | - | - | - | - | - |
| 6 | - | - | - | - | - | - |
| 7 | - | - | - | - | - | - |
| 8 | “the surgeon” | Bloods | MPADSS >8, tolerating oral intake | - | MPADSS, VAS | Telephone call |
| 9 | “the surgeon” | - | - | - | - | - |
| 10 | “the surgeon” | - | - | - | - | - |
| **Biliary disease** | | | | | | |
| 1 | - | Bloods, USS, CT | - | - | TG13, TG18, CCI | - |
| 2 | Surgical consultant | - | - | - | - | - |
| 3 | “Surgical Team” | - | Adequate analgesia and conscious level, haemodynamic stability, ability to ambulate, tolerance of oral diet | - | Clavein Dindo | Telephone call and clinic |
| 4 | - | - | - | - | Elixhauser Comorbidity Score | - |
| 5 | “the surgeon” | USS | Aldrete >9, absence of nausea and vomiting, pain control | - | Aldrete, Clavien Dindo, VAS | Home visits & clinic |
| 6 | - | Bloods | Absence of pain, bleeding or nausea and vomiting, adequate oral intake | Fever, significant pain or jaundice 7 days post-discharge | VAS | Telephone call & clinic |
| 7 | Surgical consultant | Bloods, USS | - | - | - | - |
| 8 | - | - | Aldrete score | - | Aldrete, Clavien Dindo, VAS | Telephone call |
| **Appendicitis** | | | | | | |
| 1 | - | Bloods, USS, CT | - | - | Clavien Dindo, St Antoines | Location not specified |
| 2 | Emergency department team | USS, CT | Stable vital signs, afebrile, pain control, tolerating oral intake, patient and clinician agreement, timely follow-up organised | - | EQ-5D, CCI, Alvarado score | Clinic |
| 3 | “Staff Surgeon”. | - | Adequate oral intake, full consciousness, ambulation, VAS <2, haemodynamic stability, micturition, absence of nausea and vomiting | Abdominal pain, abdominal distension, wound dehiscence, vomiting, fever | VAS | Clinic |
| 4 | “The surgeon”,, anaesthetist | USS, CT | Adequate oral intake | Nausea and vomiting, abdominal distension, fever | Clavien Dindo, St Antoines, comprehensive complication index | Clinic |
| 5 | - | Bloods, USS, CT | No complications, Aldrete criteria | - | Aldrete, Clavien Dindo, St Antoines, VAS, comprehensive complication index | Clinic |
| 6 | “the surgeon” | Bloods, USS, CT | - | - | Alvarado score, AIR score | Telephone call |
| 7 | “the surgeon” | - | Aldrete >8 | - | Aldrete, Clavien Dindo, VAS | Telephone call |
| **Diverticular disease** | | | | | | |
| 1 | ED consultant | Bloods, CT | - | - | Modified Hinchey | - |
| 2 | ED consultant/General Surgery Consultant | Bloods, CT | Uncomplicated disease, CRP <150 | Unfavourable clinical progression, signs of severity | French High Health Authority criteria | Clinic |
| 3 | - | Bloods, CT | Modified Neff grade 0 | Clinical worsening, poor symptomatic control, fever | Modified Neff, VAS | Clinic |
| 4 | Emergency department team | Bloods, CT | Pain control, absence of rebound tenderness | Progression of clinical findings, rising leukocyte or CRP levels | Modified Hinchey | Clinic |
| 5 | - | Bloods, CT | Modified Hinchey grade 0-1a, stable vital signs, afebrile, no major comorbidities, good social support, patient agreement | Abnormal observations, worsening pain, unable to maintain self-care or oral intake, generally unwell | Modified Hinchey | Home and clinic visits |
| 6 | ED consultant | Bloods, CT | Uncomplicated disease, pain and fever improved with ED management | Further CT review indicates need for ongoing investigation or management | Hinchey | GP visit |

**Key:**

AIR – Appendicitis Inflammatory Response, CCI – Charlson Comorbidity Index, CT – computed tomography scan, ED – emergency department, EQ-5D – EuroQol 5-Dimension Score, HFRS – Hospital Frailty Risk Score, IMD – Index of Multiple Deprivation, MPADSS – Modified Post-Anaesthetic Discharge Scoring System, TG13/18 – Tokyo Guidelines 2013/2018, USS – ultrasound scan, VAS – Visual Analogue Score

**Appendix 6 – Combined Cochrane Risk of Bias 2 (RoB2) Too**
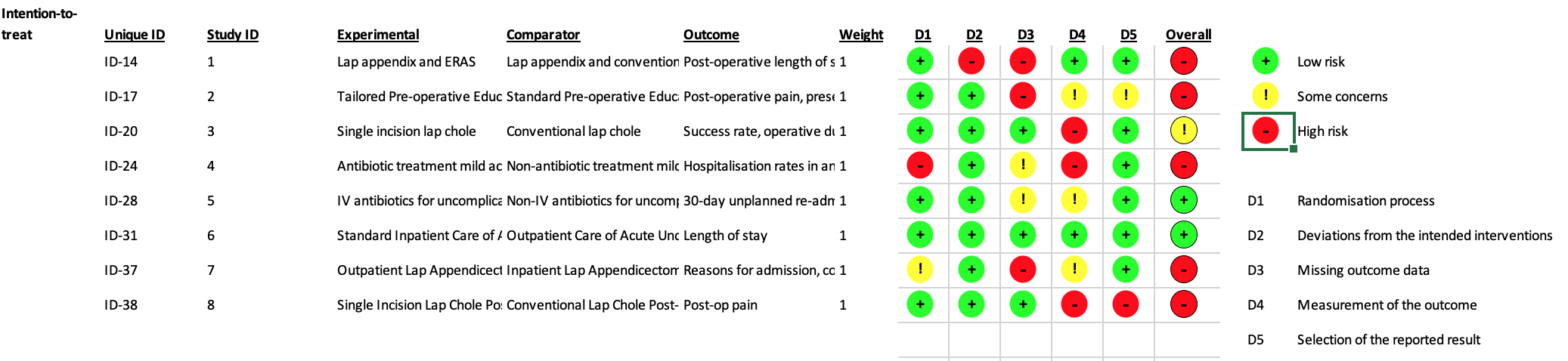
**l**
