# Supplementary material for: Use of ambulatory pathways in emergency general surgery: a systematic review
Source: BMJ Open. 2025 Sep 28;15(9):e099203. doi: 10.1136/bmjopen-2025-099203 (PMC12481336; doi:10.1136/bmjopen-2025-099203)
Supplement: Supplementary file 2 [file bmjopen-2025-099203.reviewer_comments.pdf]

PEER REVIEW HISTORY

BMJ Open publishes all reviews undertaken for accepted manuscripts. Reviewers are asked to complete a checklist review form and are provided with free text boxes to elaborate on their assessment. These free text comments are reproduced below.

ARTICLE DETAILS

Title (Provisional)

A Systematic Review of the use of Ambulatory Pathways in Emergency General Surgery

Authors

Fox, Benjamin; Walters, Mary; Pathak, Samir; Peckham-Cooper , Adam; Blencowe, Natalie S

VERSION 1 - REVIEW

**Reviewer**	**1**
**Name**	**Robin, F**
**Affiliation**	**Centre Hospitalier de l'Université de Montréal, Anesthesiology and pain medicine**
**Date**	**11-Feb-2025**
**COI**	**None**

Thank you for the opportunity to review this manuscript, which presents a literature review on the development of ambulatory surgical pathways in the context of digestive and general emergency care. This is a relevant topic, as although ambulatory surgery is growing, its integration into emergency care remains underdeveloped and highly variable across countries. This systematic review thus offers an interesting contribution to understanding and structuring these pathways.

General Remarks

The study is well conducted and follows PRISMA guidelines. However, the introduction lacks clarity and could better define the framework of the study.

The heterogeneity of the criteria reported in the included studies is a major issue, which the authors rightly highlight.

The discussion proposes relevant perspectives but could be strengthened with more concrete suggestions.

Please find below the list of my comments, which should help improve the quality of the manuscript and facilitate its understanding for the reader.

INTRODUCTION

Clarification of the study framework: The study focuses on urgent ambulatory surgical care pathways in the context of digestive (or general) pathologies. While the title of the article is explicit, it should mention the notion of general surgery. The introduction should clearly define this specific framework by explicitly mentioning the concept of "Emergency General Surgery Ambulatory Pathways" to ensure that the reader understands the scope of the study. This remark also applies to the abstract, where these terms are not explicitly mentioned, making comprehension more difficult.

(Row 26, Page 7/45):"The primary focus..." The goal of an ambulatory pathway is to ensure care with equivalent quality and safety as conventional hospitalization. Unplanned readmission is simply a potential consequence, but it is not the primary quality criterion.

METHODS

(Row 36, Page 9/45) "within a primary care setting": What does this term mean? The authors should define whether this refers to planned surgery or another type of care.

(Row 57, Page 10/45) "readmission rates": Specify whether this refers to hospitalization or return to the emergency department.

RESULTS

(Row 26, Page 12/45) "There was a total of 2,635,243 patients included across the studies, of which 1,381,359 (52%) were on ambulatory pathways." Who are the remaining 48%? Are they all "control" patients?

(Row 4, Page 13/45) Decision Making Process (DMP):

It would be interesting to specify the timing of the decision-making process: is it done before or after surgery? The specialty and grade of the clinician should be clarified: Is the final decision made exclusively by a senior, or are residents also involved? In Table 6, the authors mention 'The surgeon'—why is this in quotation marks? How does this differ from 'surgical consultant'?

(Row 25, Page 13/45) Scoring/classification systems:

Why were comorbidities and demographic data grouped together? These are two distinct concepts that could be addressed separately.

"Symptom assessment": What does this refer to exactly? Is this assessment done pre- or post-operatively?

(Row 4, Page 14/45) Re-Admission/Discharge Criteria:

Definition of readmission: The term is ambiguous. The authors should clarify whether this refers to a conversion to postoperative hospitalization.

(Row 24, Page 14/45) Follow-Up:

None of the included studies mention telehealth for patient follow-up. Given that all these studies were published since 2018, it is surprising that none mention digital tools for remote follow-up (teleconsultation, mobile application monitoring, etc.). This point could be added to the discussion.

Discussion

(Row 11, Page 16/45) "classification systems": This term could be discussed with a more descriptive phrase such as pre- and postoperative scoring systems.

(Row 43, Page 16/45) Timing of DMP: It would be interesting to specify that the timing of the decision-making process is not clearly established, and that further reflection is needed to standardize this aspect.

(Row 3, Page 17/45) "degree of pain and fatigue": This is more accurately referred to as recovery quality, and patient satisfaction could be added as a quality indicator.

(Row 13, Page 17/45):

The most important element of the discussion is defining a "core set" of criteria to structure ambulatory pathways. Instead of simply listing some criteria, the authors should propose a table summarizing all the collected criteria.

REFERENCE ISSUES

Several references are not formatted correctly (e.g., reference 17).

TABLE

Table 5: "Ongoing Management" - what exactly does this term mean?

Conclusion

This manuscript provides a valuable contribution to understanding ambulatory surgery pathways in emergency settings but could be improved with minor revision.

**Reviewer**	**2**
**Name**	**Panagiotopoulou, Ioanna**
**Affiliation**	**Cambridge University Hospitals NHS Foundation Trust, Digestive Diseases**
**Date**	**21-Feb-2025**
**COI**	**None**

This is a very well written paper in the ever developing field of ambulatory surgical care. There is a need to identify core components of the surgical ambulatory pathway, especially when developing such pathways, and it is great that the authors are looking into this area.

A few comments that may help the authors in reporting their work:

- The last sentence of the abstract concludes that the core components of ambulatory pathways can optimise patient care. I am not certain that this is a conclusion drawn from his study ie has the study proven these core components directly optimise care? So it may be best that this link is not stated in the abstract. Instead the authors are focusing on the development of pathway protocol as per title and may need expand on the development of such an ambulatory pathway protocol and clearly state and define these parts.

-p 13 42-43: Does mortality refer to cases that death occurred after decision to ambulate ie pt would not have died had they got admitted? or died as had treatment late due to the ambulatory process that was followed rather than an admission in the first place that would result in emergency surgery delivered? This would show failure of the ambulatory pathway and at what stage? Should mortality be used as an outcome for comparison esp from results from RCTs?

-p14 7-18 Again what themes were identified in complications? At what stage of the pathway did they occur? How does that change your view on the significance of each the above identified core ambulatory pathway components?

-p13: Would this theme be more accurate as "admission and discharge criteria"? When a decision is made to ambulate a surgical patient that presents as emergency, it is usually between admission or discharge to an ambulatory pathway. The term re-admission may mean failure of the ambulatory pathway and along mortality and complications should be used as outcomes measuring the safety of the ambulatory pathway.

-I think the Discussion requires more work to actually link the findings of the results and how the authors conclude as per Title that these core components of the ambulatory pathway led to the development of an ambulatory pathway protocol.

-Could you please include the risk of bias of RCTs in Results as this is reported as calculated in Methods? How did this affect your inclusion of studies and the conclusions drawn?

-Why were postoperative outcomes chosen to be the outcome of interest in Results as not all patients with RIF pain/biliary colic/hernia presentation etc will have surgery? How about mortality/re-admission/lack of follow up/time to definitive treatment?

-Would you consider a meta-analysis of outcomes within these ambulatory pathways and see how you may link them to your core components of the pathway?? eg evidence to show that follow-up must be arranged/reported after an ambulatory visit otherwise evidence show cases can lead to mortality/complication/readmission etc

---

**Reviewer**	**3**
**Name**	**Dack, Kyle**
**Affiliation**	**University of Bristol, MRC Integrative Epidemiology Unit**
**Date**	**24-Apr-2025**

COI **None**

Statistical review summary:

The methodology is reported clearly, and results are reported and interpreted accurately. I do not have any major concerns.

I do suggest the authors consider evaluating all studies – because currently the risk of bias assessment is only applied to RCTs, which leaves about 75% of studies not evaluated. There are observational assessment tools such as the NOS which might be applicable.

Abstract

1. Please add the databases which were searched to the abstract.

Introduction

2. Very clear and well written – no suggestions.

Methods

3. The search strategy section needs more details to meet the PRISMA criteria. In particular, the specific search query used for each of the four databases (could be included as a supplementary file).

4. Search strategy: it is not clear what the date range (2018 to 2023) means. Does this mean different databases were searched at different times, or is the date range for the included studies? If it is the former, this may need explaining because it is quite a large difference.

5. Study eligibility: the inclusion criteria does not include anything specific to this study – e.g. ambulatory care pathways. Please also clarify if there are any date ranges, or setting requirements (e.g. non-UK studies).

6. "All primary observational studies (i.e. case-control, cohort studies and randomised controlled trials) were included." RCTs are not observational – suggest rephrasing to "all types of observational study and randomized controlled trials" or anything similar.

7. An RoB tool is reported for RCTs – but was anything used for observational studies? There are tools available which could be relevant for case-control, cohort studies, etc, such as the Newcastle Ottawa Scale.

Results

8. Figure 1. The first box is missing data. The "reports excluded" box includes "not assessing ambulatory pathways" but this is not part of the stated exclusion criteria in the methods – this should be added so both are consistent.

9. Table 2. Using numbers for papers makes it difficult to follow – please change to the actual author names and publication dates. I suggest adding a "total" row at the end to report the N and % - this is partially reported in-text but not for all parts.

10. The remainder of the tables are clear and I do not have any major suggestions. Perhaps consider modifying the spacing / cell padding to make them more compact, and easier to read, if possible.

11. Risk of bias assessment: What were the reasons for five being at high risk of bias – were there any common criteria which were failed? More details in this section would be helpful, as would any additional assessment for the rest of the studies if an applicable tool can be found.

Discussion

12. No suggestions – all is clear and seems accurately interpreted.

VERSION 1 - AUTHOR RESPONSE

Reviewer 1 comments

Thank you for the opportunity to review this manuscript, which presents a literature review on the development of ambulatory surgical pathways in the context of digestive and general emergency care. This is a relevant topic, as although ambulatory surgery is growing, its integration into emergency care remains underdeveloped and highly variable across countries. This systematic review thus offers an interesting contribution to understanding and structuring these pathways.

General Remarks

- The study is well conducted and follows PRISMA guidelines. However, the introduction lacks clarity and could better define the framework of the study.
- The heterogeneity of the criteria reported in the included studies is a major issue, which the authors rightly highlight.
- The discussion proposes relevant perspectives but could be strengthened with more concrete suggestions.
- Please find below the list of my comments, which should help improve the quality of the manuscript and facilitate its understanding for the reader.

Reply: We thank the reviewer for their positive feedback.

Revisions: None.

1) Clarification of the study framework: The study focuses on urgent ambulatory surgical care pathways in the context of digestive (or general) pathologies. While the title of the article is explicit, it should mention the notion of general surgery. The introduction should clearly define this specific framework by explicitly mentioning the concept of "Emergency General Surgery Ambulatory Pathways" to ensure that the reader understands the scope of the study. This remark also applies to the abstract, where these terms are not explicitly mentioned, making comprehension more difficult.

Reply: We thank you for highlighting the lack of clarity with regards to the study focus. The manuscript (title, abstract and introduction) has been amended to afford greater specificity of the topic in question.

Revisions: The title has been amended to read as the following:

"A Systematic Review of ~~Emergency Surgery~~ the use of Ambulatory Pathways in Emergency General Surgery ~~Developing an Ambulatory Pathway Protocol~~"

Excerpts from the abstract **(Abstract, Pages 3 and 4)** have been amended to read as follows:

"Objectives: Whilst well-established in acute medicine, uptake of ambulatory pathways in emergency general surgery (EGS) is variable and optimal design and delivery is unclear in this context. This systematic review sought to i) appraise current EGS ambulatory pathway literature…"

"Results: Reporting of all aspects of EGS ambulatory pathways was heterogeneous…"

"Conclusions: Key components of published EGS ambulatory pathways include decision-making processes, scoring/classification systems, investigations, care escalation and discharge criteria, and follow-up. However, this information is currently inconsistently reported. Future work to identify and agree on guidelines for the 'core' components of ambulatory EGS pathways is needed, to facilitate cross-study comparisons, and crucially, provide a "gold-standard" framework for developing future ambulatory pathways."

Excerpts from the introduction **(Introduction, Page 6, paragraphs 1 and Page 7, paragraph 1)** have been amended to read the following:

"Initially implemented within emergency departments and general medicine, there is a desire to emulate this success in the emergency general surgical (EGS) setting[2]."

"When developing EGS ambulatory pathways, both patient and institutional factors require consideration and it is essential that a robust, systematic assessment of the pathway should be undertaken prior to wide implementation, with a particular focus on feasibility and safety."

2) (Row 26, Page 7/45):"The primary focus..." The goal of an ambulatory pathway is to ensure care with equivalent quality and safety as conventional hospitalization. Unplanned readmission is simply a potential consequence, but it is not the primary quality criterion.

Reply: We agree that equivocal care and safety of patients on these pathways is important and have amended text in the introduction to reflect this. We also feel that whilst re-admission rates of zero are likely to be unachievable measuring readmission rates may provide some indication of the success of the ambulatory patthways, in terms of accurate assessment of patients' suitability and provision of key discharge and readmission parameters.

Revisions: Introduction, Page 6, paragraph 2 - The authors have amended this section to read as follows:

"The primary focus of an ambulatory pathway is to ~~streamline unplanned care admission episodes allowing for~~ provide equivocal standards of care for patients outwith the hospital setting[4]. Key criteria, such as clear discharge and re-admission parameters, may help to minimise unplanned re-hospitalisation of patients on the ambulatory pathway; for example, enabling identification of "high risk" patients in whom ambulatory care may not be appropriate."

3) (Row 36, Page 9/45) "within a primary care setting": What does this term mean? The authors should define whether this refers to planned surgery or another type of care.

Reply: The "primary care setting" in this context refers to ambulatory care performed in GP practices, community pharmacies etc. This does not refer to planned surgery.

Revisions: Methods - Study Eligibility, Page 8, paragraph 3 - The authors have clarified this section to read as follows:

"Studies reporting paediatric or non-surgical populations, or ambulatory surgical care within a ~~primary~~ community care setting (such as by General Practitioners/family doctors) were also excluded. Non-English language studies were excluded."

4) (Row 57, Page 10/45) "readmission rates": Specify whether this refers to hospitalization or return to the emergency department.

Reply: Thank you for identifying the lack of clarity in this section. In this context, "readmission rates" refer to hospital re-admission.

Revisions: Methods - Outcomes, Page 10, paragraph 1 - The authors have clarified this section to read as follows:

"Outcomes including complications, care escalation to hospital, and mortality were collected."

5) (Row 26, Page 12/45) "There was a total of 2,635,243 patients included across the studies, of which 1,381,359 (52%) were on ambulatory pathways." Who are the remaining 48%? Are they all "control" patients?

Reply: Thank you for identifying this. The remaining 48% were not necessarily controls, but rather those who did not meet the criteria to be included on the specific ambulatory pathway.

Revisions: Results - Characteristics of Included Studies, Page 11, paragraph 2 - The authors have clarified this section of the results to read as follows:

“A total of 2,635,243 patients were included across the studies, of which 1,381,359 (52%) were on ambulatory pathways (the remainder did not meet the criteria for ambulatory management).”

6) (Row 4, Page 13/45) Decision Making Process (DMP): It would be interesting to specify the timing of the decision-making process: is it done before or after surgery? The specialty and grade of the clinician should be clarified: Is the final decision made exclusively by a senior, or are residents also involved? In Table 6, the authors mention 'The surgeon'—why is this in quotation marks? How does this differ from 'surgical consultant'?

Reply: Thank you for commenting on this. To clarify your point regarding the decision-making process; the decision being made was not in regard to surgery, but whether a patient met the criteria and was thought to be deemed suitable for care on the ambulatory pathway. Only 6 studies commented on specific timing of when the SDM made the decision to ambulate, and this has now been included in the revised manuscript.

The grade of the clinician is described in the “Decision making process” paragraph within the Ambulatory Pathway Components section of the results, but we agree that further clarity could be provided as to their speciality. The term “The Surgeon” is in quotation marks because this is what some papers used to describe the senior decision-maker. No clarity was provided as to the speciality, grade or seniority of the clinician in these included papers.

Revisions: Results - Decision Making Process, Page 12, paragraph 2 and 3 - We have altered the main manuscript to include the speciality of the clinician as well as grade:

24 (56%) reported the specialty and grade of clinician acting as SDM. SDM was most commonly a consultant/attending (EGS/General Surgery = 6, ED = 3, Anaesthetics = 2, whilst in 3 studies the SDMs were EGS/General Surgery residents (n=3).

Six of the 43 studies commented on specific timing of ambulation. In two, ambulation occurred post-operatively (within 6-8 hours), one within 8 hours of treatment in the Emergency Department, one following an ultrasound scan within 24hrs, one within 72hrs following joint consultation between ED and General Surgery, and one following a scheduled appointment with the EGS team (the timing of which was not specified).

7) (Row 25, Page 13/45) Scoring/classification systems: Why were comorbidities and demographic data grouped together? These are two distinct concepts that could be addressed separately. "Symptom assessment": What does this refer to exactly? Is this assessment done pre- or post-operatively?

Reply: Thank you for highlighting this. The authors recognise that these two concepts should not be grouped together and have now separated them. The scoring system being referred to for “symptom assessment” is the Visual Analogue Score, which is a subjective measure of pain scoring. This assessment can be done at any time in the pre- or post-operative course, and the implementation of this varied depending on the ambulatory pathway and type of pathology.

Revisions: Results - Scoring/Classification Systems, Page 12, paragraph 4 - The authors have revised the scoring/classification system paragraph as follows:

"Across these, 17 different systems were used, for a variety of purposes: disease severity/diagnostic workup (n=6), comorbidities (n=3), patient demographics (n=1), suitability for ambulation/discharge (n=3), post-operative outcomes (n=3) and symptom assessment (n=1)."

8) (Row 4, Page 14/45) Re-Admission/Discharge Criteria: Definition of readmission: The term is ambiguous. The authors should clarify whether this refers to a conversion to postoperative hospitalization.

Reply: Thank you for highlighting this. Again, due to the heterogeneity of the ambulatory pathways, and as a result of reviewer comments below, we have altered this term to "care escalation", which was defined as any inpatient stay after discharge on an ambulatory pathway. Dependent on the pathway, this may have been pre- or post-operatively. This does not refer to prolonged hospital stay following an operative procedure. The authors have sought to clarify this distinction in the methods in order to provide greater clarity throughout the paper.

Revisions: Methods - Outcomes, Page 10, paragraph 1 - The authors have clarified the above in the methods section:

"Outcomes including complications, care escalation to hospital, and mortality were collected. Care escalation was defined as any inpatient stay after discharge onto an ambulatory pathway. Dependent on the pathway, this may have occurred pre- or post-operatively, or may not have been associated with an operative procedure."

9) (Row 24, Page 14/45) Follow-Up: None of the included studies mention telehealth for patient follow-up. Given that all these studies were published since 2018, it is surprising that none mention digital tools for remote follow-up (teleconsultation, mobile application monitoring, etc.). This point could be added to the discussion.

Reply: Apologies that this is not clearer. The authors would recommend referring to **Table 4**, which includes the follow-up location, personnel undertaking follow-up, format of follow-up, time point of follow-up and number of follow-up appointments. This shows that 9 of the 43 studies used telehealth appointments as part of their follow-up.

Revisions: Results - Follow-up, Page 13, paragraph 4 - The authors have clarified the above in the results section:

"Follow-up was described in 29 papers, with location (n=29), time-points (n=26), personnel (n=16), and the form that the follow-up took (n=23) reported variably. Most commonly, follow-up took place in an ambulatory clinic (n=21), or remotely using telehealth (n=9) and was clinician-led (n=-14) using history and examination (n=23)."

10) (Row 11, Page 16/45) "classification systems": This term could be discussed with a more descriptive phrase such as pre- and postoperative scoring systems.

Reply: Thank you for making this point. In this context, classification systems are used to categorize patients, procedures, or surgical complications based on various factors. They may help standardize assessments, facilitate communication, and to determine disease severity. However, the challenge with using the description 'pre and post-operative scoring systems' is many emergency surgical patients do not undergo operative intervention (e.g. diverticulitis). In addition, the heterogeneity of each ambulatory pathway included in the study was reflected in the 17 different scoring systems used, making it difficult to accurately describe them collectively. Thus, we feel that keeping the phrase broad allows for an appreciation of the huge variation in types and functions of scoring systems used.

Revisions: None.

11) (Row 43, Page 16/45) Timing of DMP: It would be interesting to specify that the timing of the decision-making process is not clearly established, and that further reflection is needed to standardize this aspect.

Reply: We agree that this is a key point and attention should be offered to this critical component of ambulatory pathways. We have as such included a short sentence in the discussion to allude to this.

Revisions: Discussion, Page 16, paragraph 1 - The authors have amended the first paragraph of the discussion to read as follows:

"Amongst the 43 included papers, reporting of these components was variable ~~and heterogeneous~~ and there was little agreement on which outcomes should be measured (and how). Key components of ambulatory pathways - for example, 6 studies commented on the timing of the decision to ambulate a patient - were not considered, leaving ambiguity as to the optimal time for patients to enter an ambulatory pathway."

12) (Row 3, Page 17/45) "degree of pain and fatigue": This is more accurately referred to as recovery quality, and patient satisfaction could be added as a quality indicator.

Reply: We agree that this recovery quality is a more appropriate term, and recognise the importance of patient satisfaction as a quality indicator.

Revisions: Discussion, Page 17, paragraph 2 - The authors have amended the excerpt from paragraph 3 of the discussion to the following:

"Previous reviews have attempted to evaluate the commonly reported quality indicators in ambulatory surgery[16]. These indicators have often been divided into "pre-operative" (which include same-day cancellation and cancellation of procedures after patient arrival) "peri-operative" (including clinical information provided) and "post-operative" (which include 24hr post-operative clinical state, recovery quality (degree of pain and fatigue) and patient satisfaction)[16]. However, these quality indicators are not applicable to all ambulatory

pathways, given that many EGS conditions (e.g. diverticulitis, pancreatitis) do not require surgery. This study has promoted the development of more universally recognised quality indicators: decision-making processes, classification systems, investigations, care escalation/discharge criteria, and follow-up. We propose building on these elements into a blueprint for the "gold-standard" development of an ambulatory pathway..."

13) (Row 13, Page 17/45): The most important element of the discussion is defining a "core set" of criteria to structure ambulatory pathways. Instead of simply listing some criteria, the authors should propose a table summarizing all the collected criteria.

Reply: Thank you for your suggestion. We have included a table **(Table 5)** at the end of the manuscript to summarise this. However, it is important to note that the degree of heterogeneity amongst ambulatory pathways makes it challenging to set specific components that every pathway must adhere to. Further research needs to be undertaken to identify if further key components exist that are not already mentioned in the findings of this paper.

Revisions: Table 5, page 28 - The authors have included a new Table at the end of the manuscript to summarise suggested "core" requirements for creation of an ambulatory pathway.

Table 5 – Summary of Suggested "Core Set" of Descriptors for Construction of Ambulatory Pathway

SDM	Scoring and classification systems	Investigations	Care Escalation/Discharge criteria	Follow-up
Agreed role of SDM within ambulatory pathway<br><br>Agreed level of experience sufficient to undertake role of SDM<br><br>Clear aims of SDM with regards to ambulation. This could include whether the SDM is also	Use of a minimum number of risk severity scoring systems to compliment decisions regarding ambulation. For example, use of HAPS score in acute pancreatitis<br><br>Clear cut-offs for when ambulation of a patient is	Appropriate and timely use of imaging such as USS or CT, including whether this is used to determine suitability for ambulation.<br><br>Follow-up investigations including repeat blood tests as mandatory part of ongoing	Clear, defined criteria for care escalation of patients following regular clinic follow-up.<br><br>Consider use of physical, biochemical and radiological criteria to ensure a full clinical picture is used to make	Use of novel resources e.g. remote virtual monitoring wards to identify early those patients who are at high-risk and who may require non-ambulatory care.<br><br>Consider early, regular follow-up of patients on ambulatory pathways, and

the final decision maker in the ambulatory process.	considered too high-risk.<br><br>Built in use of “clinical judgement” to compliment severity scoring, to ensure we are not just “treating numbers”.	review of patients on an ambulatory pathway.<br><br>A second pathway for those patient’s who’s imaging requires non-ambulatory management, to ensure the same standards of care are maintained for both ambulatory and non-ambulatory patients.	decisions regarding ambulation.<br><br>Discharge criteria that risk-stratify patients to ensure that patients who are ambulated are suitable for ambulation.<br><br>“Complications resulting in re-admission should be documented and scoring systems (such as Clavien-Dindo) should be used to risk-stratify these complications. These can form a basis for identifying areas for improvement within the pathway itself.”	clear pathways for surgical intervention should this be necessary (e.g. use of “hot” gallbladder lists for ambulated patients who require laparoscopic cholecystectomy).

14) Several references are not formatted correctly (e.g., reference 17).

Reply: Thank you for highlighting this. We have edited the relevant references so that they meet the recommended reference guidelines.

Revisions: Bibliography, Page 22-23 - The authors have amended several references as per below:

“7) Menon N, Griffin J , Kumar S, Jackson I, McWhinnie D. Reflections on Ambulatory Pathways in the Post-Covid Era. Ambul Surg. 2020;1(1):49–52.

8) Anderson, I. THE FUTURE OF EMERGENCY GENERAL SURGERY ~~THE FUTURE OF EMERGENCY GENERAL SURGERY~~:A joint document Key Recommendations. 2015.
17) NHS England. Emergency Care Data Set v 2.1 [Internet]. 2015 [cited 2024 Sep 16]. Available from: https://www.england.nhs.uk/wp-content/uploads/2015/12/ecds-v2-1.pdf"

15) Table 5: "Ongoing Management" - what exactly does this term mean?

Reply: We included the term "Ongoing Management" in reference to the relevant general surgical pathology in question. This included the need for further antibiotics or planning surgical intervention.

Revisions: Results - Follow-up, Page 13, paragraph 4 - The authors have clarified the term "Ongoing Management" in the results section as per below:

"Follow-up was described in 29 papers, with location (n=29), time-points (n=26), personnel (n=16), and the form that the follow-up took (n=23) reported variably. Most commonly, follow-up took place in an ambulatory clinic (n=21), or remotely using telehealth (n=9) and was clinician-led (n=-14) using history and examination (n=23)." 6 studies also described "Ongoing Management", which included continued antibiotic prescriptions and surgical planning."

Conclusion:
This manuscript provides a valuable contribution to understanding ambulatory surgery pathways in emergency settings but could be improved with minor revision.

Reply: We thank the reviewer for their support.

Reviewer 2 comments

General Remarks
This is a very well written paper in the ever-developing field of ambulatory surgical care. There is a need to identify core components of the surgical ambulatory pathway, especially when developing such pathways, and it is great that the authors are looking into this area.

Reply: We thank the reviewer for their encouragement.

1) The last sentence of the abstract concludes that the core components of ambulatory pathways can optimise patient care. I am not certain that this is a conclusion drawn from his study ie has the study proven these core components directly optimise care? So it may be best that this link is not stated in the abstract. Instead the authors are focusing on the development of pathway protocol as per title and may need expand on the development of such an ambulatory pathway protocol and clearly state and define these parts.

Reply: We agree that whilst optimising patient care is a key outcome of a well established ambulatory pathway, it has not been demonstrated in this study. We have amended the

conclusions drawn in the abstract to expand on the key pathway components identified, and future research suggestions.

Revisions: Abstract, Page 4, paragraph 3 - The authors have revised the conclusions from the abstract to read as follows:

"Conclusions: Key components of published EGS ambulatory pathways include decision-making processes, scoring/classification systems, investigations, care escalation and discharge criteria, and follow-up. However, this information is currently inconsistently reported. Future work to identify and agree on guidelines for the 'core' components of ambulatory EGS pathways is needed, to facilitate cross-study comparisons, and crucially, provide a "gold-standard" framework for developing future ambulatory pathways."

Discussion, Page 17, paragraph 2 - The authors have also revised the discussion to read as follows:

"We propose building on these elements into a blueprint for the "gold-standard" development of an ambulatory pathway, to include an overarching "core set" of ambulatory pathway components, that should always be included and reported. Facilitating an agreed structure would help to promote safety and improve the quality and consistency of future pathways, facilitating cross-study comparisons between centres and pathways."

2) p 13 42-43: Does mortality refer to cases that death occurred after decision to ambulate ie pt would not have died had they got admitted? or died as had treatment late due to the ambulatory process that was followed rather than an admission in the first place that would result in emergency surgery delivered? This would show failure of the ambulatory pathway and at what stage? Should mortality be used as an outcome for comparison esp from results from RCTs?

Reply: Thank you for this excellent point. The mortality refers to deaths from patients who were allocated to ambulatory pathways. It is difficult to draw conclusions as to whether these patients would have survived if the decision not to ambulate had been made, as only 8 of the 43 included studies were RCTs that directly compared ambulatory vs non-ambulatory patients. In addition, only 24 papers recorded mortality, with 21 of these reporting a mortality of 0%. Of the 3 papers that reported mortality rates, these were 0.3%, 0.004% and 0.003% respectively - none of these papers described the cause of death and reported mortality only. We don't believe that mortality was a sufficient measure of success or failure of the ambulatory pathways and as such it was not a primary focus of the paper.

Revisions: Results - Follow-up, Page 14, paragraph 2 - The authors have added details on the mortality reporting measures in the results section as per below:

"Mortality was recorded in 24 papers, with 21 reporting a mortality rate of 0 and the remaining three <0.5%. None of the 3 papers that reported a mortality rate greater than 0 described the cause of the mortality."

3) -p14 7-18 Again what themes were identified in complications? At what stage of the pathway did they occur? How does that change your view on the significance of each the above identified core ambulatory pathway components?

Reply: Again this is an excellent point. There was a general lack of consistency and transparency in the reporting of complications. 34 studies reported complication rates ranging from 0 to 48.5%. Although the remaining 8 studies reported "complications" no further details were provided (including the frequency with which they occurred). None of the papers reported when the complications occurred during the pathway. Part of future direction in the study could include a clear set of reporting guidelines on when complications occur, in which part of the pathway, and any identified steps to minimise these complications.

Revisions: Discussion, Page 16, paragraph 1 and Table 5, page 28 -The authors have included a comment on the complications and lack of clarity of reporting of these in the discussion, to better link the results as you suggest:

"Reporting of complications (n=34) and mortality (n=24), either pre- or post-operatively, and when these occurred within each specific ambulatory pathway, were also not adequately described. In addition, only 8 studies reported adequate care escalation and discharge criteria, leaving significant room for error when making decisions regarding a patient's course within a specific ambulatory pathway. There is, therefore, an urgent need for a clear, structured approach to detailing the essential components of a pan-pathology surgical ambulatory pathway."

We have also added comments on the need for transparency regarding complications as part of our "core set" of ambulatory pathway criteria in **Table 5**:

"Complications resulting in re-admission should be documented and scoring systems (such as Clavien-Dindo) should be used to risk-stratify these complications. These can form a basis for identifying areas for improvement within the pathway itself."

4) p13: Would this theme be more accurate as "admission and discharge criteria"? When a decision is made to ambulate a surgical patient that presents as emergency, it is usually between admission or discharge to an ambulatory pathway. The term re-admission may mean failure of the ambulatory pathway and along mortality and complications should be used as outcomes measuring the safety of the ambulatory pathway.

Reply: Thank you for highlighting this point. This theme is essential because any robust ambulatory pathway should have criteria which maintain patient safety in case of the need for escalation of treatment. We agree that use of the term "re-admission" is misleading, and would suggest that instead, we use the term "care escalation" - which recognises the need to identify patients on an ambulatory pathway that may require subsequent admission. In this case, only 9 studies reported care escalation criteria as part of their pathway. Lack of clear reporting of these criteria offers an opportunity for us to better design future pathways, but it does not allow us to draw accurate conclusions as to their safety.

Revisions: The authors have altered the theme to "care escalation and discharge criteria" throughout the manuscript.

5) I think the Discussion requires more work to actually link the findings of the results and how the authors conclude as per Title that these core components of the ambulatory pathway led to the development of an ambulatory pathway protocol.

Reply: We thank you for highlighting this point. Whilst we have summarised the core components from the results section in the discussion, we agree these should be better described and discussed in the discussion.

Revisions: Discussion, Page 16, paragraph 1 and Page 17, paragraph 2 - The authors have added in further detail to paragraphs 1 and 3 of the discussion:

"This review summarised current evidence regarding the ambulatory management of EGS conditions ~~ambulatory pathways for general surgery patients~~ and identified five key pathway components ~~involved in ambulation~~: decision-making processes, classification systems, investigations, care escalation/discharge criteria, and follow-up. Amongst the 43 included papers, reporting of these components was variable ~~and heterogeneous~~ and there was little agreement on which outcomes should be measured (and how). Key components of ambulatory pathways - for example, 6 studies commented on the timing of the decision to ambulate a patient - were not considered, leaving ambiguity as to the optimal time for patients to enter an ambulatory pathway. Reporting of complications (n=34) and mortality (n=24), either pre- or post-operatively, and when these occurred within each specific ambulatory pathway, were also not adequately described. In addition, only 8 studies reported adequate care escalation and discharge criteria, leaving significant room for error when making decisions regarding a patient's course within a specific ambulatory pathway. There is, therefore, an urgent need for a clear, structured approach to detailing the essential components of a pan-pathology surgical ambulatory pathway."

"Previous reviews have attempted to evaluate the commonly reported quality indicators in ambulatory surgery[16]. These indicators have often been divided into "pre-operative" (which include same-day cancellation and cancellation of procedures after patient arrival), "peri-operative" (including clinical information provided), and "post-operative" (which include 24hr post-operative clinical state, recovery quality (degree of pain and fatigue) and patient satisfaction)[16]. However, these quality indicators are not applicable to all ambulatory pathways, given that many EGS conditions (e.g. diverticulitis, pancreatitis) do not require surgery. This study has promoted the development of more universally recognised quality indicators: decision-making processes, classification systems, investigations, care escalation/discharge criteria, and follow-up. We propose building on these elements into a blueprint for the "gold-standard" development of an ambulatory pathway, to include an overarching "core set" of ambulatory pathway components, that should always be included and reported."

6) Could you please include the risk of bias of RCTs in Results as this is reported as calculated in Methods? How did this affect your inclusion of studies and the conclusions drawn?

Reply: We have already included a short summary of the Risk of Bias assessment at the end of the "Results" section, and included the formal assessment in "Appendix 6" of the "Supplementary Materials" document. 5 of the 8 RCTs were considered "high-risk" of bias, so conclusions drawn from these studies should be interpreted with caution.

Revisions: The authors have added further detail to the Risk of Bias assessment summary (**Results - Risk of Bias Assessment, Page 15, paragraph 2)**:

"Of the 8 RCTs, five were considered at "high-risk" of bias (commonest areas of concern were missing outcome data and outcome measurement), with 1 having "some concerns" and 2 being "low-risk".

and to the discussion section **(Discussion - Page 18, paragraph 2):**

"The authors also recognise that whilst a ROB assessment tool was used for the randomised control trials, quality assessment was not undertaken for other study designs. Given most of the included RCTs were at high risk of bias, we anticipated that the quality of the other studies would also be poor."

7) Why were postoperative outcomes chosen to be the outcome of interest in Results as not all patients with RIF pain/biliary colic/hernia presentation etc will have surgery? How about mortality/re-admission/lack of follow up/time to definitive treatment?

Reply: Thank you for this excellent point. Post-operative outcomes were one of many outcomes of interest which included re-admission rates and mortality (please also see "Outcomes" paragraph in methods). Whilst as you rightly state that not all studies will have surgery as part of their ambulatory protocol, for those who did, there was a huge variety in the reporting of these complications, highlighting another way in which developing a 'core set' of information for ambulatory surgery pathways is likely to be very useful.

Revisions: Discussion, Page 17, paragraph 2 - The authors have included a comment to address this in the discussion section of the paper:

These indicators have often been divided into "pre-operative" (which include same-day cancellation and cancellation of procedures after patient arrival), "peri-operative" (including clinical information provided), and "post-operative" (which include 24hr post-operative clinical state, recovery quality (degree of pain and fatigue) and patient satisfaction)[16]. However, these quality indicators are not applicable to all ambulatory pathways, given that many EGS conditions (e.g. diverticulitis, pancreatitis) do not require surgery".

8) Would you consider a meta-analysis of outcomes within these ambulatory pathways and see how you may link them to your core components of the pathway?? eg evidence to show that follow-up must be arranged/reported after an ambulatory visit otherwise evidence show cases can lead to mortality/complication/readmission etc

Reply: Thank you for this excellent suggestion. Due to the heterogeneity of emergency general surgical conditions that this study encompasses, and the heterogeneity of reporting

quality, we feel that a meta-analysis is likely to offer limited benefit and would create challenges with drawing accurate conclusions from such an analysis. However, we agree that this is one direction that our future research on the topic may take.

Revisions: Discussion, Page 18, paragraph 2 - The authors have included a comment on future implementation of a meta-analysis in the discussion:

"Second, the review intended to give an overarching view of ambulatory surgical care and therefore included many different surgical conditions, which may have influenced the lack of consistency in results, although 'matched' comparisons of papers reporting pathways for the same condition suggest this not to be the case. The variety in surgical conditions included in this review meant that performing a meta-analysis would have had limited value".

Reviewer 3 comments

General Remarks

- The methodology is reported clearly, and results are reported and interpreted accurately. I do not have any major concerns.
- I do suggest the authors consider evaluating all studies – because currently the risk of bias assessment is only applied to RCTs, which leaves about 75% of studies not evaluated. There are observational assessment tools such as the NOS which might be applicable.

1) Abstract - Please add the databases which were searched to the abstract.

Reply: Thank you for this comment; this has also been noted by the editor and we have amended the abstract to meet the recommendations for formatting the abstract.

Revisions: Abstract, Page 3, paragraph 3 - The authors have amended the abstract to include the data sources as follows:

"Data Sources: PubMed, Embase, Medline, and Cochrane Library, from 5th December 2018 to 5th December 2023 inclusive."

2) Introduction - Very clear and well written – no suggestions.

Reply: Thank you for the feedback!

Revisions: None.

3) Methods - The search strategy section needs more details to meet the PRISMA criteria. In particular, the specific search query used for each of the four databases (could be included as a supplementary file).

Reply: Rather than include specific details in the search strategy section itself, we have included granular detail pertaining to each specific search query in the Supplementary Materials Section Appendix 1: Search Strategy. We have also included search strategy details relating to PROSPERO registration as a new Appendix 2. This PRISMA Flowchart is also included in Figure 1 for convenience.

Revisions: Supplementary Materials document – Appendix 1 and 2. **Figure 1 – Separate document.**

4) Search strategy: it is not clear what the date range (2018 to 2023) means. Does this mean different databases were searched at different times, or is the date range for the included studies? If it is the former, this may need explaining because it is quite a large difference.

Reply: We apologise for the lack of clarity regarding this. The date range refers to the date range for the included studies.

Revisions: Methods - Search Strategy and Study Identification, Page 8, paragraph 2 - The authors have amended this section to the following:

"Systematic searches - including terms for ambulatory care, general surgery and specific surgical conditions - were undertaken in PubMed, Embase, Medline, and Cochrane Library, to include studies dating from 5th December 2018 to 5th December 2023 inclusive (See **Appendix 1**)".

5) Study eligibility: the inclusion criteria does not include anything specific to this study – e.g. ambulatory care pathways. Please also clarify if there are any date ranges, or setting requirements (e.g. non-UK studies).

Reply: Thank you for highlighting this. We have expanded this section to clarify further inclusion/exclusion criteria.

Revisions: Methods - Study Eligibility, Page 8, paragraph 3 - The authors have amended this section to read as follows:

"Primary observational studies (i.e. case-control, cohort studies) and randomised control trials providing data about EGS ambulatory pathways were included. Case series and conference abstracts were excluded due to the high likelihood of incomplete data".

6) "All primary observational studies (i.e. case-control, cohort studies and randomised controlled trials) were included." RCTs are not observational – suggest rephrasing to "all types of observational study and randomized controlled trials" or anything similar.

Reply: Apologies for not making this clearer. This has now been amended.

Revisions: Methods - Study Eligibility, Page 8, paragraph 3 - The authors have now amended this sentence to read as follows:

“Primary observational studies (i.e. case-control, cohort studies) and randomised control trials providing data about EGS ambulatory pathways were included”.

7) An RoB tool is reported for RCTs – but was anything used for observational studies? There are tools available which could be relevant for case-control, cohort studies, etc, such as the Newcastle Ottawa Scale.

Reply: Unfortunately, no RoB tool was used for the other studies.

Revisions: Discussion, Page 18, paragraph 2 - The authors have included a comment in the limitations paragraph of the results section to address this:

“The authors also recognise that whilst a ROB assessment tool was used for the randomised control trials, quality assessment was not undertaken for other study designs. Given most of the included RCTs were at high risk of bias, we anticipated that the quality of the other studies would also be poor.”

8) Results - Figure 1. The first box is missing data. The “reports excluded” box includes “not assessing ambulatory pathways” but this is not part of the stated exclusion criteria in the methods – this should be added so both are consistent.

Reply: Thank you for highlighting this. We have added further to the methods to reflect this.

Revisions: Methods - Study Eligibility, Page 8, paragraph 3 - The authors have further edited the “Study Eligibility” section of the methods as follows:

“Studies reporting paediatric or non-surgical populations, or ambulatory surgical care within a ~~primary~~ community care setting (such as by General Practitioners/family doctors) were also excluded. Non-English language studies were excluded.”

9) Table 2. Using numbers for papers makes it difficult to follow – please change to the actual author names and publication dates. I suggest adding a “total” row at the end to report the N and % - this is partially reported in-text but not for all parts.

Reply: We thank you for this point. We have changed the table to include author names and publication dates. We have also added a total row to the end of the Table. However, due to the size of the table the authors were required to include this in the supplementary materials document.

Revisions: Supplementary Materials – Appendix 4.

10) The remainder of the tables are clear and I do not have any major suggestions. Perhaps consider modifying the spacing / cell padding to make them more compact, and easier to read, if possible.

Reply: Thank you for this point. Where possible, we have formatted the tables to ensure that they are as easy to read as possible.

Revisions: Formatting edits have been made to all tables in the manuscript.

11) Risk of bias assessment: What were the reasons for five being at high risk of bias – were there any common criteria which were failed? More details in this section would be helpful, as would any additional assessment for the rest of the studies if an applicable tool can be found.

Reply: We have included the Combined Cochrane Risk of Bias Assessment Tool as Appendix 3, which includes the criteria in which papers were considered at high risk of bias. 3 papers were at high risk of bias in criteria D3 (Missing outcome data) and D4 (Measurement of the outcome). Unfortunately no additional assessments were carried out.

Revisions: Results - Risk of Bias Assessment, Page 15, paragraph 2 - The authors have added a short sentence to add to the Risk of Bias summary:

"Of the 8 RCTs, five were considered at "high-risk" of bias (commonest areas of concern were missing outcome data and outcome measurement), with 1 having "some concerns" and 2 being "low-risk".

12) Discussion - No suggestions – all is clear and seems accurately interpreted.

Reply: Thank you!

Revisions: None.

VERSION 2 - REVIEW

**Reviewer**	**1**
**Name**	**Robin, F**
**Affiliation**	**Centre Hospitalier de l'Université de Montréal, Anesthesiology and pain medicine**
**Date**	**29-Jul-2025**
**COI**	

I would like to thank the authors for their comprehensive and thoughtful revisions. All my comments have been fully addressed, and the manuscript has significantly improved in clarity and quality. I have no further suggestions and support the publication of this revised version.
